# Global Stress Responses Identify the Functionally Divergent Regulators Required for *Candida auris* Commensalism and Pathogenicity

**DOI:** 10.1002/EXP.20240482

**Published:** 2025-11-11

**Authors:** Chaoyue Xu, Wanxing Xu, Yushun Yuan, Xiaoqing Chen, Ouyang Mo, Zhe Yin, Xinhua Huang, Yuanyuan Wang, Lingfei Hu, Wenwen Xue, Yun Zou, Luyao Zhang, Kunlin Li, Yueru Tian, Jihong Liu, Sichu Xiong, Lei Wu, Yanmei Dong, Guangsheng Chen, Yuping Zhang, Zili Zhou, Ming Guan, Xiaotian Huang, Zhiyi He, Lin Zhong, Lingbing Zeng, Pei Hao, Xiaoqi Zheng, Changbin Chen, Ning‐Ning Liu, Dongsheng Zhou

**Affiliations:** ^1^ Joint Laboratory for Biomedical Research and Pharmaceutical Innovation Unit of Pathogenic Fungal Infection and Host Immunity Key Laboratory of Molecular Virology and Immunology Shanghai Institute of Immunity and Infection Chinese Academy of Sciences Shanghai China; ^2^ Nanjing Advanced Academy of Life and Health Nanjing China; ^3^ State Key Laboratory of Systems Medicine for Cancer Center for Single‐Cell Omics School of Public Health Shanghai Jiao Tong University School of Medicine Shanghai China; ^4^ Department of Mathematics Shanghai Normal University Shanghai China; ^5^ State Key Laboratory of Pathogen and Biosecurity Academy of Military Medical Sciences Beijing China; ^6^ Department of Laboratory Medicine Huashan Hospital North Shanghai Medical College Fudan University Shanghai China; ^7^ Department of Gastroenterology and Hepatology Characteristic Medical Center of the Chinese People's Armed Police Force Tianjin Key Laboratory of Hepatopancreatic Fiberosis and Molecular Diagnosis & Treatment Tianjin China; ^8^ Department of Respiratory and Critical Care Medicine The First Affiliated Hospital of Guangxi Medical University Nanning Guangxi China; ^9^ Department of Medical Microbiology School of Medicine Nanchang University Nanchang China; ^10^ National Clinical Research Center for Infectious Disease The Third People's Hospital of Shenzhen Shenzhen Guangdong China; ^11^ Department of Laboratory Medicine The First Affiliated Hospital of Nanchang University Nanchang China

**Keywords:** *Candida auris*, Comparative transcriptomic analysis, Pan‐genome, Stress response

## Abstract

Given its global distribution and high transmissibility in the environment, *Candida auris* poses a serious threat to global public health. However, the underlying mechanisms of its adaptive strategies remain poorly understood. Here we delineate the pan‐genome structures of 1,306 representative *C. auris* isolates collected from 28 countries. In addition to the clade‐related genetic diversity and highly variable pan‐genomes, we identify the key regulatory modules and genes specific to *C. auris* in response to 32 different host microenvironment‐mimicking stresses. Through comparative analysis with evolutionarily close fungal relatives, we uncover both shared and species‐specific transcriptional responses in *C. auris*. Intriguingly, our results reveal a distinct pathogenic role for the conserved iron regulon in this species. Unexpectedly, we also identify an evolutionarily divergent functional role for *RIM101* in regulating both pathogenicity and commensalism of *C. auris*. Mechanistically, the high‐affinity glucose transporters were found to enhance the tolerance to alkaline stress through alleviation of *RIM101*‐dependent glucose repression in the host microenvironment. These findings provide mechanistic insights into the evolutionarily divergent adaptive strategies in both commensalism and virulence of the emerging critical priority fungal pathogen, *C. auris*.

## Graphical abstract

*Candida auris* is an emerging multidrug‐resistant fungal pathogen that poses a serious global health threat. In our study, we comprehensively delineate both the pan‐genomic and transcriptomic features of *C. auris*. Intriguingly, our findings reveal an unexpected alkaline pH‐dependent regulation required for *C. auris* virulence and commensalism mediated by *RIM101*. Mechanistically, the high‐affinity glucose transporters were found to enhance the tolerance of *C. auris* to alkaline stress through alleviation of *RIM101*‐dependent glucose repression. These findings should aid in the development of precision therapeutic strategies targeting evolutionarily divergent transcriptional regulators in fungal pathogens such as *C. auris*.

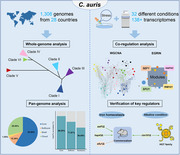

## 1 Introduction

Human fungal pathogens pose a great threat to human health due to the emergence of new species, the expanded epidemiological regions, and the increased prevalence of antifungal resistance. Documented in over 50 countries across six continents since 2009 [1, 2, 3], *C. auris* was recognized as the most alarming agent for candidemia nowadays and categorized into six major phylogenetic clades [4, 5]. Different clades in distinct geographical locations exhibit substantial genetic diversity and phenotypic variation. Since the first report of a clinical isolate of *C. auris* (BJCA001) in China in 2018, the number of *C. auris* isolates has now increased to 312, covering 10 provinces and 18 hospitals as of December 2023 [3, 6]. Recently, *C. auris* has been included in the critical priority group of the fungal priority pathogens list released by WHO [7], due to its rising pan‐drug resistance and documented ability to persist in nosocomial settings. This landmark inclusion represents the first global initiative to systematically prioritize fungal disease research, aiming to guide scientific efforts toward addressing this emerging public health threat [8].

Unraveling the multifaceted interplay between host immune responses, microbial virulence determinants, and environmental cues shapes disease outcome and highlights the complexity of host‐pathogen‐environment interactions in fungal pathogenesis [9, 10, 11, 12, 13, 14]. Recently, *C. auris* was isolated from the salt marsh and sandy beach in the Indian Ocean [15]. The existence of *C. auris* was also found to be in diverse harsh environments, including high temperatures, high salt, and clinical decontamination procedures [15, 16, 17]. However, there is limited evidence regarding the mechanism of host‐pathogen interactions during *C. auris* infection in response to the dynamic changes of host microenvironments [18]. Our comprehensive understanding of *C. auris* virulence remains impeded by the paucity of functional characterization studies elucidating the molecular mechanisms underlying its stress responses. This knowledge gap significantly limits our capacity to develop effective countermeasures against this rapidly evolving fungal pathogen, underscoring the urgent need for mechanistic investigations into its adaptive strategies.

Here, we characterized both clade‐specific genetic diversity and dynamic pan‐genomes, and identified core and species‐specific regulatory modules that underpin its pathogenesis to *C. auris*. Notably, we uncover evolutionarily conserved yet functionally divergent roles for the alkaline pH‐dependent regulatory network in mediating *C. auris* virulence and commensalism, governed by a conserved transcription regulator *RIM101*. Mechanistic investigation revealed that the High‐affinity Glucose Transporters (HGT) enhance the tolerance to alkaline stress in *C. auris* by alleviating *RIM101*‐dependent repression of glucose absorption and utilization within the host microenvironment. These findings advance our understanding of pathogenic and commensal determinants in *C. auris* and highlight evolutionarily divergent transcription regulators as potentially precision therapeutic strategies against fungal infections.

## 2 Results

### 2.1 The Global Distribution and Pan‐Genome Structure of the *C. auris* Isolates

**FIGURE 1:**
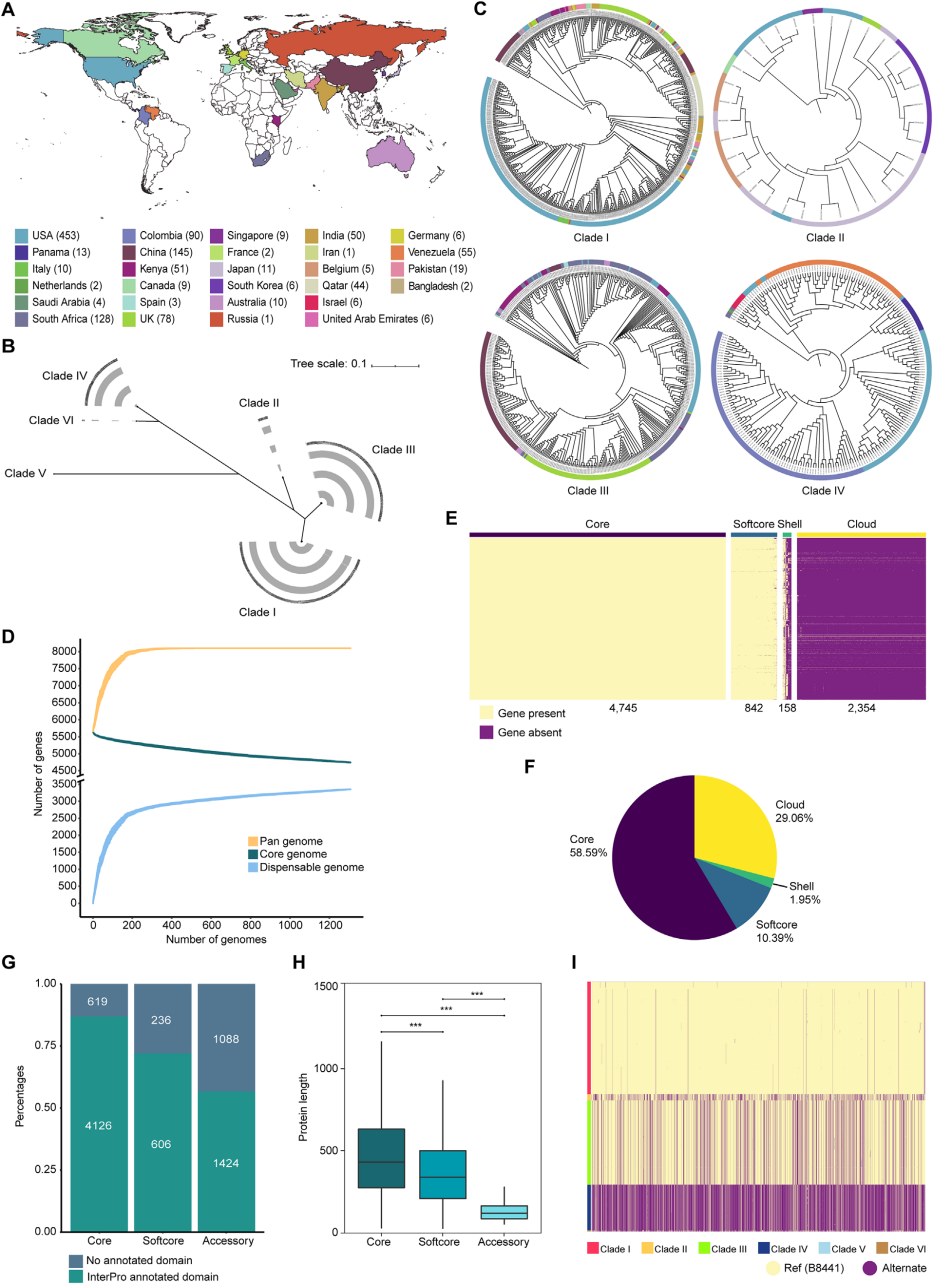
Pan‐genomic analysis of globally distributed *C. auris* strains. (A) Global distribution of 1,306 sequenced *C. auris* isolates from 28 countries. (B) The phylogenetic tree constructed from whole‐genome sequences clustered *C. auris* isolates into six major clades. (C) Detailed phylogenetic trees of clades I to VI, with countries denoted by distinct colors. (D) Pan‐genome size of *C. auris* varies as the number of included genomes rises. The thickness of the curve represents the 99% confidence interval. (E) The presence/absence matrix was constructed for 8,099 gene clusters identified from 1,306 *C. auris* genomes. The pan‐genome was subdivided into core genes (found in all strains), soft core genes (found in >95% of strains), shell genes (found in 5%–95% of strains), and cloud genes (found in <5% of strains), according to the frequency of gene presence across all strains. (F) Composition elements of the pan‐genome in *C. auris*. (G) Count and fraction of core, softcore, and accessory genes harboring an annotated InterPro domain. (H) The length of proteins encoded by core, softcore, and accessory genes, with all *p*‐values being significantly below 2.22 × 10^−16^. (I) Distribution of SNPs sites in different clades (SNP sites = 208,524). The statistical significance in (H) was determined using two‐sided Mann–Whitney *U*‐test with Bonferroni's correction; ***p *<* 0.001.

To gain an overall insight into the global distribution, we first conducted phylogenetic analysis of 1,306 *C. auris* isolates from 28 countries, including two whole genome sequences from Jiangxi province (JXCA001, Fluconazole resistant) and Peking city (Peking1, BJCA001, Fluconazole susceptible) in China (Figure 1A and Table S1). We identified six primary clades (Figure 1B,C), aligning with previous studies [5, 19, 20, 21, 22]. Specifically, 589 isolates were categorized into Clade I across 16 countries, including the USA and China. The two local isolates were classified into Clade I, along with strain B8441 (the reference strain), albeit from a distinct branch. Clade II was present in six countries, such as Japan, Clade III in eight countries, including South Africa, Clade IV in seven countries, such as Colombia, Clade V exclusively in Iran, and Clade VI in Singapore and Bangladesh (Figure 1B,C). Isolates from each country were identifiable to at least one of the six clades, while those from the USA were classified into four clades. This diverse clade representation might be due to potential contributors such as global traveling and the persistence of *C. auris* in both colonized patients and healthcare settings [23].

We next applied pan‐genome analysis to map and explore genetic feature variations of *C. auris* isolates belonging to the six different clades. The average genome size of our assemblies was 12.05 Mb, with an average contig number of 526 and the mean N50 of 152,160 base pairs (Tables S2 and S3). We found 98.55% of single‐copy orthologs were fully assembled by BUSCO. With the continuous inclusion of the genome sequences, the gene numbers approached saturation, indicating a “closed” pan‐genome (Figure 1D). As proposed by Barber et al. [24], we categorized the total 8,099 genes into 4,745 core genes, 842 softcore genes, 158 shell genes, and 2,354 cloud genes (Figure 1E,F and Table S4). In particular, 2,516 novel genes were identified. Further analysis revealed that 86.95% of the core genes contained at least one annotated InterPro domain compared with 71.97% of the softcore genes and 56.69% of the accessory genes (Figure 1G). The protein sequences of core genes were significantly longer than those of softcore or accessory genes (Figure 1H). Gene ontology (GO) annotations revealed significant enrichment of functional categories related to protein binding, ATP binding, ATPase activity, and oxidoreductase activity in the *C. auris* core genome. In contrast, the accessory genome demonstrated enrichment for transmembrane transporter activity, proteolysis, and carbohydrate metabolic processes. Consistent with these findings, Pfam domain analysis also identified a high proportion of protein kinase domains in the core genome and enrichment of major facilitator superfamily (MFS) and sugar transporter domains in the accessory genome (Figure S1 and Table S5). Taken together, these results indicate distinct functional repertoires between core and accessory genomic compartments, underpinning functional divergence between conserved metabolic pathways and the adaptive transport system.

Furthermore, we analyzed the single nucleotide variations (SNVs) within each clade (Figure 1I), revealing distinct evolutionary signatures. Clade I displayed the highest genomic conservation relative to the reference genome, while Clade IV exhibited the highest genomic divergence with widespread mutational distributions. Functional enrichment analysis identified Clade I‐specific enrichment in conserved metabolic pathways and cell wall biogenesis processes, whereas Clade IV demonstrated significant enrichment in signal transduction cascades and cellular stress responses (Table S5). Notably, 549 isolates exhibit clear resistance to fluconazole, with these resistant strains predominantly clustered into Clade I and Clade III. Using the fluconazole‐susceptible reference strain B8441, we applied stringent filtering criteria to identify single‐nucleotide polymorphisms (SNPs) present in >95% of susceptible isolates but <5% resistant isolates. These analyses identified key candidate genes associated with fluconazole resistance, including *HGT7*, *PGA7*, and *FAS2* (Table S6), which were differentially expressed under fluconazole stress in *C. albicans* [25, 26]. Specifically, *HGT7* (high‐affinity glucose transporter 7) encodes a putative glucose transporter belonging to the MFS and exhibits significant upregulation in *C. albicans* grown in YPD medium under standard yeast growth conditions [27]. The expression of *FAS2* (alpha subunit of fatty acid synthetase) and *PGA7* (GPI‐linked hyphal surface antigen) was induced under hypoxic conditions in *C. albicans* [28, 29]. Intriguingly, stringent filtering criteria failed to identify any SNPs that were predominantly present in 95% of the resistant isolates while being infrequent (<5%) in the susceptible strains. Finally, we performed a genome‐wide association study (GWAS) to identify target genes associated with fluconazole resistance. This analysis was conducted specifically on Clade IV due to its balanced distribution of resistance phenotype. We identified 23 genes, 10 of which have orthologs in *C. albicans*. Among them, missense mutations in five genes (e.g., *HXK2*) may affect carbon metabolism, transcriptional regulation, and cell morphology. Upstream mutations in eight genes could enhance antioxidant capacity (e.g., *SOD1*), signal transduction (e.g., *LTP1* and *HBR1*), protein homeostasis (e.g., *DOA4*), stress response (e.g., *SBP1*), and cell morphology regulation (e.g., *HOF1*). Synonymous mutations in 10 genes are involved in membrane transport, lipid metabolism, and epigenetic modifications (e.g., *SMF3*, *TEF1*, and *FAD2*). These findings link the fluconazole resistance to potential mechanisms related to drug transport, energy metabolism, membrane permeability, protein stability, and stress responses, and of course, further experimental validation is required. Taken together, the pan‐genome analysis revealed that the core genome is enriched in annotated domains and proteins with longer amino acid sequences, and that SNPs‐associated genes contribute to fluconazole resistance through multiple mechanism. Importantly, these genomic observations highlight the genetic basis underlying the adaptability of *C. auris* to diverse environments.

### 2.2 Transcriptional Adaptability of *C. auris* in Response to Diverse Environmental Stresses

**FIGURE 2:**
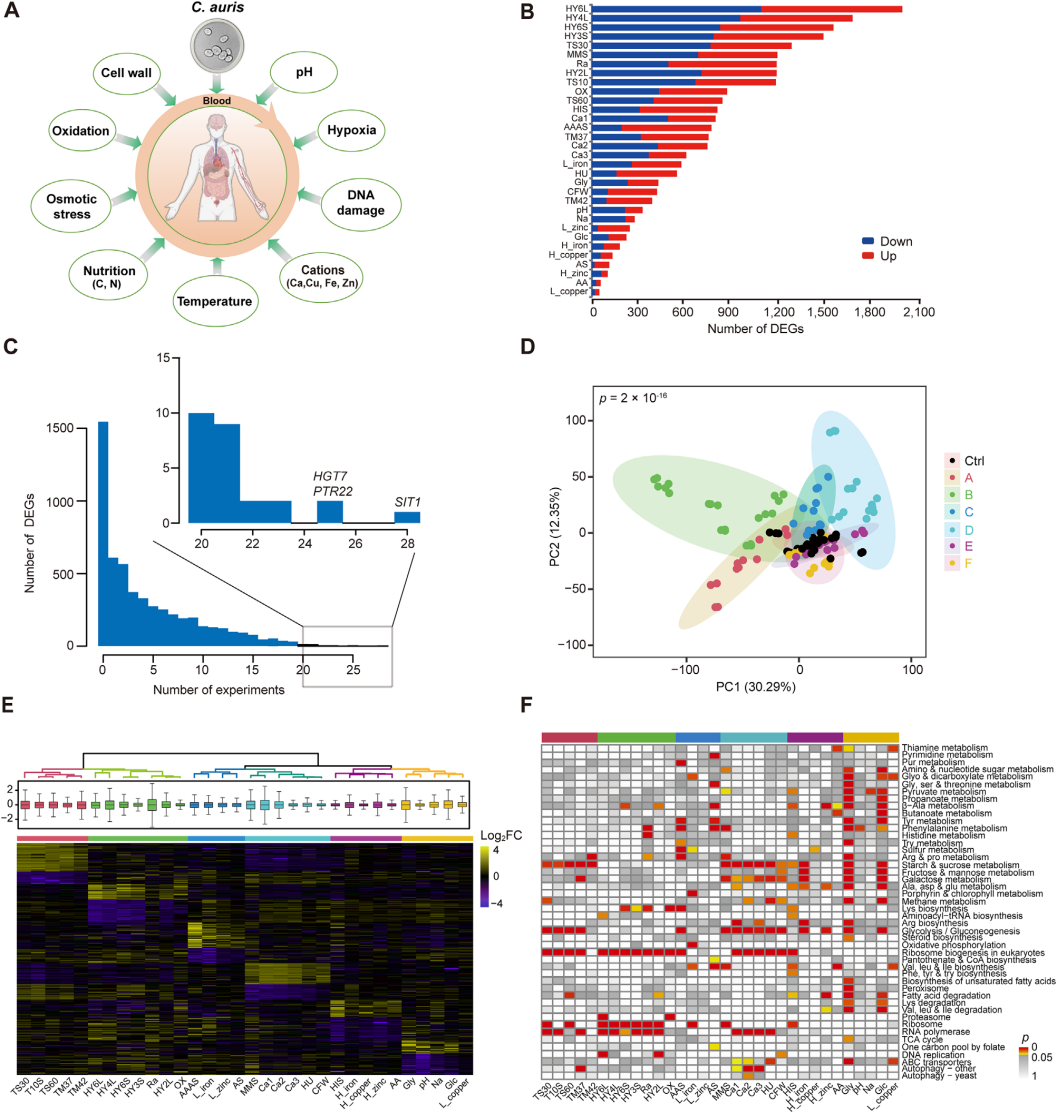
Transcriptional profiling of *C. auris* under 32 stress conditions. (A) Schematic illustration depicting the experimental conditions. (B) Shown is the number of DEGs under various stress conditions, blue signifies down‐regulation, while red represents up‐regulation. (C) Distribution of the number of DEGs detected concurrently across multiple stresses. Those DEGs found in 20 or more conditions are highlighted. (D) PCA plot illustrating six groups of stress conditions determined through hierarchical clustering. Each dot represented an individual sample, and the black dots signify the control samples. (E) Heatmap illustrating the log‐fold changes in gene expression for all stress conditions when compared to the control samples. The same hierarchical clustering method employed in (D) was utilized here. (F) The significance of gene set enrichment analysis (GSEA) of the KEGG pathway under each stress condition. The data presented in (B,F) served as representatives of three independent experiments. *p*‐value in (D) was calculated by an ANOVA test conducted on the first principal component. *p*‐values in (F) were adjusted by Benjamini–Hochberg method.

Similar to the other fungal pathogens, *C. auris* also exhibits changes in transcriptional regulation in response to environmental stresses. Given the strong relationship between the genomic classification and gene expression profiles, we conducted a transcriptomic analysis to determine the effect of genomic changes on gene expression levels, and more importantly, to identify key regulatory factors involved in fungal stress adaptation. We assessed the transcriptional adaptability of *C. auris* in response to a total of 32 distinct environmental stresses, of which mimic the diverse host microenvironments and include nine host‐associated factors such as temperature [30, 31], DNA damage [32, 33, 34, 35, 36], nutrient [37, 38], pH [39, 40, 41, 42], and cations [40, 43, 44, 45] (Figure 2A). A total of 138 RNA sequencing data was collected, with an average of ~48 million sequencing reads per sample after filtering. We identified a total of 3,854 differentially expressed genes (DEGs) and found that the number of DEGs varied significantly across different conditions (Table S7). For example, under the hypoxic conditions (HY6L, HY4L, HY6S, HY3S), we identified the top four stress‐responsive genes, while under low copper conditions, only 49 DEGs were detected (Figure 2B). Furthermore, 2,682 genes are differentially expressed in multiple stress conditions (≥3 different stresses), and three of them (*B9J08_002480*, the BLASTN hit of *HGT7*; *PTR22*; and *B9J08_001487*, the BLASTN hit of *SIT1*) showed similar expression patterns under more than 25 stress conditions (Figure 2C). These three genes were significantly down‐regulated under more than half of these conditions. Interestingly, *PTR22* (oligopeptide transporter involved in uptake of di‐/tripeptides), the gene found to be repressed by *RIM101* under alkaline conditions in *C. albicans* [46], was up‐regulated in *C. auris*. Hierarchical clustering analysis partitioned all samples into six distinct clusters, which was further confirmed by the principal component analysis (PCA) (Figure 2D). Each group covered at least four different stress conditions (Figure 2E). In particular, DEGs of Group 5 (‐HIS, ‐AA, high iron, high copper, and high zinc) were significantly enriched in oxidoreductase activity and aspartate kinase activity, key components of central carbon metabolism. In contrast, those of Group 6 (Gly, Glc, pH, Na, and low copper) were predominantly associated with transmembrane transporter activity, implying their distinct roles in environmental adaptation and stress tolerance.

Furthermore, we identified pathways uniquely enriched in DEGs under distinct environmental perturbations. For instance, under exposure to H_2_O_2_, DEGs were significantly enriched in RNA metabolism and ribosome biosynthesis pathways (Figure 2F and Figure S3A). This transcriptional signature differed from previously characterized stress response profiles in *C. albicans*, which typically involve membrane transporters, cell surface‐related, and REDOX pathways [47]. Under conditions such as CFW (Calcofluor White), Rapa (Rapamycin), and Na (NaCl), DEGs were found to be enriched in metabolic pathways, but no differences in the Mitogen‐Activated Protein Kinase (MAPK) pathway, which contradicts previous reports in *C. albicans* [48] (Figure 2F and Figure S3B). This discrepancy may be attributed to post‐translational modifications (phosphorylation) by the MAPK pathway occurring at the protein level rather than mRNA level. Under amino acid starvation conditions, including ‐AA‐AS, ‐AA, ‐AS, and ‐HIS, the pathways were consistently enriched in amino acid metabolism pathways such as glycolysis/gluconeogenesis, β‐alanine metabolism, and starch and sucrose metabolism. Finally, under conditions of high iron and copper exposure, *C. auris* showed significant enrichment in Arg biosynthesis, fructose, mannose, and galactose metabolic pathways. Notably, high iron condition elicited more robust enrichment of these pathways compared to high copper stress (Figure 2F and Figure S2C,D). Collectively, our transcriptomic results identified distinct stress adaptation strategies, conserved stress‐responsive genes, and condition‐specific pathway enrichments operating in *C. auris*, and provided critical insights into its pathogenicity mechanisms under the challenge of diverse host environmental stresses.

### 2.3 Shared and Specific Transcriptional Responses in Comparison With the Other Fungal Species

**FIGURE 3:**
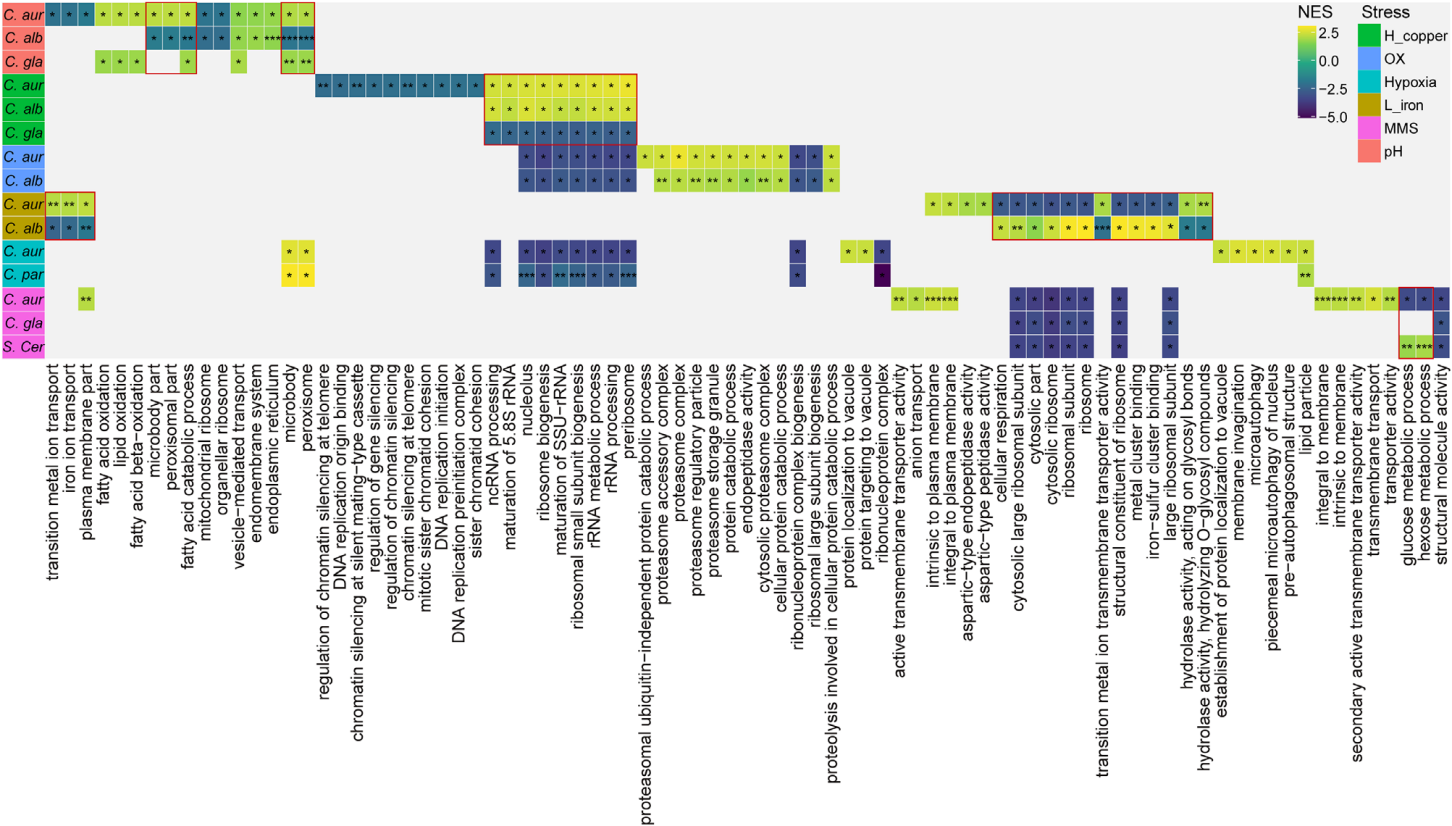
Comparative analysis of the transcriptional profiles of DEGs between *C. auris* and other fungal species under varied experimental conditions. Heatmap depicting the normalized enrichment score (NES) of diverse GO terms in different strains under six stress conditions: alkaline (pH: red), high copper (H_copper: green), H_2_O_2_ (OX: blue), low iron (L_iron: deep yellow), hypoxia (cyan), and MMS (pink). NES was computed by applying the GSEA method. Colors with a lighter shade signify up‐regulation, and colors with a darker shade denote down‐regulation. *p*‐values were calculated by permutation test and adjusted by Benjamini–Hochberg method. *p < 0.05; **p < 0.01; ***p < 0.001.

To investigate the evolutionary conservation and divergence of transcriptional adaptation mechanisms across fungal species, we performed Gene Set Enrichment Analysis (GSEA) using KEGG and GO gene sets. This integrative analysis compared *C. auris* transcriptional responses with orthologous datasets from *Candida albicans*, *Candida glabrata*, *Candida parapsilosis*, and *Saccharomyces cerevisiae* under six stress conditions, including alkaline, H_2_O_2_, high copper, low iron, methyl methanesulfonate (MMS) and hypoxia (Figure 3 and Table S8). Our approaches combined de novo RNA‐seq data with publicly available transcriptomic datasets to identify conserved and species‐specific pathways underlying stress tolerance. For example, under alkaline conditions, *C. auris* and *C. glabrata* showed significant changes of gene expression in top 20 GO terms (10 positively and 10 negatively enriched), including fatty acid and catabolic process, microbody and peroxisome, which was in contrary to *C. albicans*. Under high copper condition, both *C. auris* and *C. albicans* showed upregulations in rRNA maturation and ribosome biogenesis, whereas *C. glabrata* exhibited the opposite. Notably, *C. auris* and *C. albicans* exhibited concordant transcriptional responses to hydrogen peroxide (H_2_O_2_), whereas their regulatory patterns diverged significantly under iron‐limiting conditions. Specifically, *C. auris* displayed upregulation of transition metal ion transport alongside marked downregulation of cellular respiration processes in low‐iron environments. Under hypoxic condition, *C. auris* and *C. parapsilosis* exhibited similar transcriptional responses, particularly in the lipid particle biogenesis pathway. In contrast, *C. auris* and *S. cerevisiae* displayed divergent regulatory patterns under MMS stress, with *C. auris* demonstrating significant downregulation of glucose and hexose metabolic processes compared to *S. cerevisiae*. Interestingly, *C. auris* and *C. albicans* showed opposing transcriptional responses under iron‐limiting and alkaline conditions, suggesting species‐specific regulatory mechanisms may underpin their differential adaptive strategies. Additionally, long non‐coding RNA (lncRNA) annotation of *C. auris* transcriptome identified 447 novel transcripts, representing potential species‐specific regulatory elements that may contribute to its unique phenotypic plasticity (Table S9). Together, comparative transcriptomic analysis revealed *C. auris*‐specific regulatory divergence in stress response pathways, while maintaining conserved core stress response frameworks shared with other fungal species. These findings highlight both evolutionary conservation and lineage‐specific development in transcriptional adaptation mechanisms involving the complex interplay between conserved regulatory networks and species‐specific genetic determinants.

### 2.4 Regulatory Network Inference Identified Key Modules and Genes Under Different Stress Conditions

**FIGURE 4:**
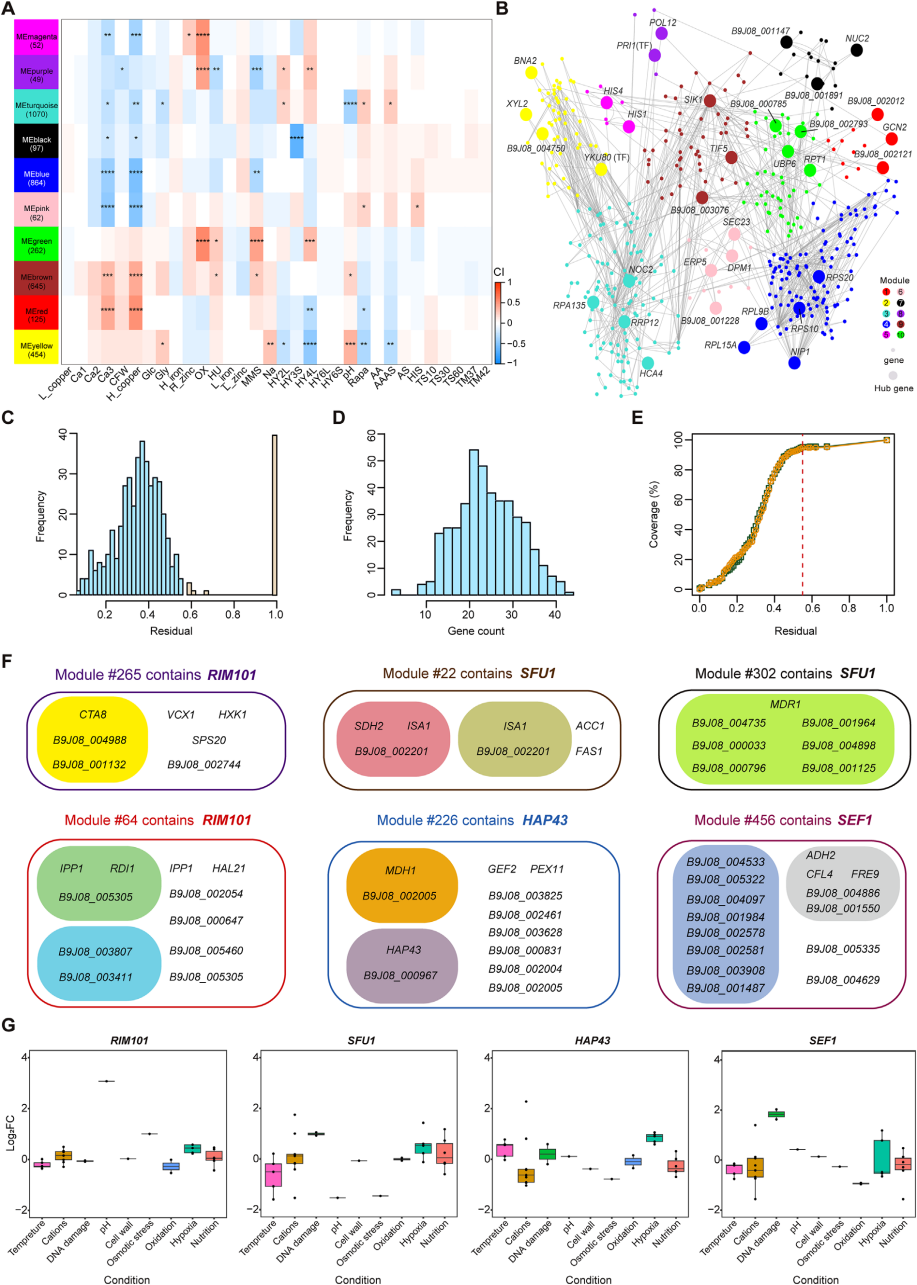
WGCNA and EGRIN analyses identify key TFs in stress responses of *C. auris*. (A) Heatmap of the correlations between module eigengenes and conditions. The number of genes in each module is indicated on the left. (B) Co‐expression network with nodes representing genes colored by modules and edges between genes with correlated expression profiles. Each module was associated with specific gene functions, such as metabolism of cofactors and vitamins (blue, *p* = 0.019), transport and catabolism (brown, *p* = 0.037), folding, sorting and degradation (green, *p* = 4.82 × 10^−39^), amino acid metabolism (magenta, *p* = 7.24 × 10^−13^), glycan biosynthesis and metabolism (pink, *p* = 1.36 × 10^−4^), replication and repair (purple, *p* = 3.03 × 10^−12^), cell growth and death (red, *p* = 0.58), translation (turquoise, *p* = 1.24 × 10^−8^), carbohydrate metabolism (yellow, *p* = 5.32 × 10^−10^), and oxidative phosphorylation (black, *p* = 1.33 × 10^−38^). The largest circle represents the hub gene, and the relationships of all the genes are connected by lines. (C) The distribution of the residual values is presented for the 541 co‐regulated gene modules that were detected. Among them, 421 modules having a residual value greater than 0.55, which are depicted in blue, were classified as being of high quality. (D) Distribution of the count of genes within the high‐quality gene modules. (E) Coverage of all 5,417 genes and 346 TFs (we tentatively define that any protein with a DNA binding site might act as a potential TF) by EGRIN modules at various residual thresholds. The red dashed line denotes the 0.55 residual threshold cutoff. (F) EGRIN modules containing TFs *HAP43*, *SFU1*, *SEF1*, and *RIM101*. In each module, genes are enriched in distinct functional terms. (G) The expression profiles of TFs *HAP43*, *SFU1*, *SEF1*, and *RIM101*. The samples are categorized according to different conditions. Statistical significance in (A) was determined using unpaired Student's *t* test and adjusted by Benjamini–Hochberg method; *p < 0.05; **p < 0.01; ***p < 0.001; ****p < 0.0001. *p* values of gene enrichment analysis in (B,F) were determined using Hypergeometric test and adjusted by Benjamini–Hochberg method.

To identify the specific gene modules that exhibited similar expression patterns associated with certain stress phenotypes, we performed Weighted Gene Co‐expression Network Analysis (WGCNA) [49] and classified all potential DEGs into 10 different modules. The module eigengenes (MEs), which can be used as representatives of the gene expression profiles from modules, were enriched in specific KEGG pathways. Intriguing results were observed based on the correlations of stress conditions with overrepresented modules (Figure 4A). In general, the MEgreen module (folding, sorting, and degradation) and MEmagenta module (amino acid metabolism) were positively correlated with the H_2_O_2_ stress condition, whereas the MEblack module (oxidative phosphorylation) was negatively correlated with the hypoxia condition. Moreover, the regulatory network and hub genes of each module were identified based on the MEs and intra‐module connectivity, respectively. Among the hub genes identified, *PRI1* in MEpurple module has been reported to regulate DNA replication and repair in the budding yeast *S. cerevisiae* [50], and *GCN2* in MEred module was found to be nonessential for cell growth under amino acid starvation in *C. albicans*, in contrast to its importance in *S. cerevisiae* [51]. These findings support the notion that these hub genes may also play key roles in the stress responses of *C. auris*.

To explore the regulation patterns of stress‐correlated genes, we constructed the Environment and Gene Regulatory Influence Network (EGRIN) model using cMonkey2 [52]. This approach infers modules that are co‐regulated rather than simply co‐expressed. The EGRIN network effectively organized 5,386 out of 5,417 genes into 541 modules. After filtering, we identified 421 modules, accounting for 77.8% of the total modules (Figure 4C). The high‐quality modules effectively captured transcriptional regulation of 5,373 genes (99.2% of the total 5,417, including 346 TFs) (Figure 4D,E).

Modules containing the TFs such as *HAP43*, *SFU1*, *SEF1*, and *RIM101* attracted our attention, since they were key regulators involved in the fungal responses to iron [53, 54] and alkaline conditions [55, 56]. The genes highlighted in selected modules were considered as significant co‐regulated genes of the four TFs (Figure 4F and Table S10). Particularly, in modules #265 and #64, genes co‐regulated with *RIM101* were enriched in sequence‐specific DNA binding transcription factor activity, cytoplasm, and ubiquitin‐dependent protein catabolic process. In modules #22 and #302, genes co‐regulated with *SFU1* were enriched in the iron–sulfur cluster binding pathway, fatty acid biosynthetic process, and transmembrane transport process. In module #22, genes co‐regulated with *HAP43* were enriched in carboxylic acid metabolic process and sequence‐specific DNA binding transcription factor activity. In module #456, genes co‐regulated with *SEF1* were enriched in transmembrane transporter activity and oxidoreductase activity. Furthermore, we assessed the expression levels of the four transcription factors (TFs) under diverse stress conditions. *RIM101* showed the highest expression level under alkaline conditions, and *SFU1* and *SEF1* were upregulated under DNA damage conditions, and *HAP43* was upregulated under hypoxia conditions (Figure 4G). Notably, we found consistency between the specific functional modules identified by WGCNA and the TFs associated regulatory modules determined by cMonkey2. This consistency was particularly evident in key signaling pathways involved in protein degradation (MEgreen and *RIM101*), amino acid metabolism (MEmagenta and *SFU1*), and oxidative phosphorylation (MEblack and *SEF1*). These findings suggest that co‐expression analysis (WGCNA) and co‐regulation analysis (EGRIN) complement each other, working together to uncover the molecular mechanisms underlying the stress response in *C. auris*.

### 2.5 The Distinct Role of Conserved Iron Regulon in Virulence and Commensalism Between *C. auris* and *C. albicans*

**FIGURE 5:**
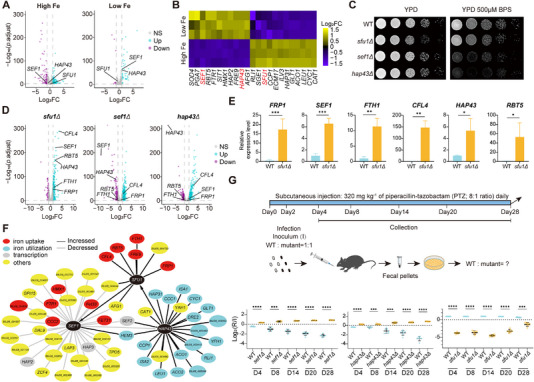
The iron circuit containing TFs *SFU1*, *SEF1*, and *HAP43* is required for *C. auris* virulence and commensalism. (A) DEGs of *C. auris* when comparing the high iron conditions with the iron‐limited conditions are presented. Purple plots depict genes that are significantly down‐regulated in the experimental group as compared to the control group. Blue graphs signify genes that are significantly upregulated, while gray graphs indicate genes showing no significant difference between the two conditions. (B) The expression changes of genes regulated by iron in *C. auris* under low‐iron conditions. A heatmap illustrates the genes involved in iron metabolism among DEGs when iron is limited. Notably, three essential TFs (*HAP43*, *SFU1*, and *SEF1*) are marked in red. (C) The growth phenotypes of the gene knockout strains of *HAP43* and *SEF1* on the iron‐limited culture medium, as well as the growth outcome of the gene knockout strain of *SFU1* on a high copper medium, were observed after 48 h. (D) DEGs in the *sef1Δ* and *hap43Δ* mutant strains grown under the iron‐limited culture medium, as well as those in the *sfu1Δ* mutant grown under the iron‐rich YPD medium. (E) qRT‐PCR validates the RNA‐Seq data for the representative *SFU1* gene targets. These genes were analyzed in both *C. auris* WT and *sfu1Δ* mutant strains, which were cultivated in the iron‐rich YPD medium. (F) Shown is the regulatory network of TFs *HAP43*, *SEF1*, and *SFU1* in *C. auris* with respect to their downstream genes. A black line denotes an increase in gene expression, while a gray line signifies a decrease. Target genes associated with iron uptake are shaded in red, those related to iron‐utilization are colored blue, TFs are depicted in gray, and all others genes are shown in yellow. (G) Commensalism experiment of *C. auris*. C57BL/6 mice were subjected to oral gavage with 1:1 mixture of the WT strain and each of the three knockout strains (*sef1Δ*, *hap43Δ* or *sfu1Δ*). The abundance of each strain present in the inoculum (I) and after recovery from fecal pellets (R) was determined by qPCR. Vertical dotted lines in (A) and (D) denote the cutoff of two‐fold change, and the horizontal dotted line represents the significance cutoff at *p* = 0.05. Data are representatives of at least three independent experiments. ns, no significance; *p < 0.05, **p < 0.01, ***p < 0.001, ****p < 0.0001 by unpaired Student's *t* test (E and G).

*C. auris*, an emerging nosocomial pathogen, demonstrates a remarkable capacity to colonize abiotic surfaces in healthcare settings despite stringent disinfection procedures. This resilience could be attributed to species‐specific transcriptional programs governing environmental adaptation. Current functional annotation of *C. auris* remains heavily dependent on orthologous genes identified in *C. albicans*, despite significant phylogenetic divergence between these two species. Under iron‐limited conditions, we used 23 previously identified genes associated with iron absorption and utilization in *C. albicans* [44] and generated a heatmap depicting their expression dynamics (Figure 5A,B). We focused on three TFs—*HAP43*, *SEF1*, and *SFU1*—that serve as core regulators contributing to iron homeostasis in *C. albicans*. Transcriptomic analysis revealed significant upregulation of *HAP43* and *SEF1* under low‐iron stress, accompanied by downregulation of *SFU1* (Figure 5A). This regulatory pattern coincided with activation of specific iron uptake genes and repression of iron utilization genes (Figure 5B), mirroring previously characterized iron‐responsive network in *C. albicans*.

To mechanistically characterize the roles of these three iron‐responsive TFs (*SFU1*, *SEF1*, and *HAP43*) in *C. auris*, we generated knockout mutants for each TF (Figure S4A). Under iron‐limiting conditions, both *hap43Δ* and *sef1Δ* mutants, but not *sfu1Δ* mutant, exhibited significant growth defects on solid medium (Figure 5C). Moreover, transcriptional analysis revealed upregulation of six genes (*FRP1*, *FTH1*, *CFL4*, *SEF1*, *HAP43*, and *RBT5*) in *sfu1Δ* mutant under low iron stress (Figure 5D,E) and consistent downregulation of a number of genes associated with iron absorption and utilization in *sef1Δ* mutant. Conversely, *hap43Δ* mutant showed hyperactivation of iron uptake genes. Comparative regulatory network analysis uncovered striking conservation of downstream targets regulated by *HAP43*, *SEF1*, and *SFU1* between *C. auris* and *C. albicans*, despite their evolutionary divergence. Intriguingly, this regulatory architecture differed significantly from *S. cerevisiae*, another haploid fungus and also a non‐pathogenic yeast counterpart. *AFT2* is a key transcriptional activator in *S. cerevisiae* that induces the expression of iron‐uptake genes under iron‐depletion conditions. Knocking out *AFT2* in *S. cerevisiae* leads to defective growth in iron‐restricted medium [57, 58]. Interestingly, while *C. albicans AFT2* homolog has been implicated in iron metabolism; its deletion does not elicit growth defects under iron‐restricted conditions [57]. To explore this evolutionary divergence, we generated a knockout mutant of the *C. auris* ortholog of *AFT2* and performed growth assays under iron‐limiting conditions. No significant growth defects were observed in *hap43Δ* mutant of *C. auris* (Figure S4B), consistent with findings in *C. albicans*. These data suggest that functional divergence in iron‐responsive TFs may represent a key adaptive advantage distinguishing pathogenic *Candida* species (e.g., *C. auris* and *C. albicans*) from non‐pathogenic yeast counterparts like *S. cerevisiae*.

Finally, integrative analysis revealed a cohesive regulatory network encompassing DEGs involved in iron absorption and utilization that are regulated by *SFU1*, *SEF1*, and *HAP43* (Figure 5F). Functional validation using the established *Drosophila* model of *Candida* infection indicated virulence defects in all three TF knockout mutants compared to wild type (WT), with *sef1Δ* mutant exhibiting significant attenuation (Figure S4C). To assess intestinal commensalism, a murine gastrointestinal colonization model was employed. Over a 28‐day period, *sef1Δ* and *hap43Δ* mutants displayed competitive fitness advantages, whereas *sfu1Δ* mutant showed impaired colonization (Figure 5G). These results suggest an opposing role of *SFU1* in promoting symbiosis versus *SEF1* and *HAP43* in inhibiting commensalism. Interestingly, comparative analysis with *C. albicans* revealed contrasting regulatory dynamics: while *C. albicans* pathogenicity requires both *SEF1* and *HAP43* activities, gut colonization is positively regulated by *SFU1* and *SEF1*, and negatively by *HAP43* [44, 59]. Collectively, these results demonstrated distinct roles of the evolutionally conserved iron regulons on the virulence and commensalism between *C. auris* and *C. albicans*.

### 2.6 Loss of *rim101* Favors Both Commensalism and Virulence of *C. auris* by Alleviating Glucose Repression in Alkaline Condition

**FIGURE 6:**
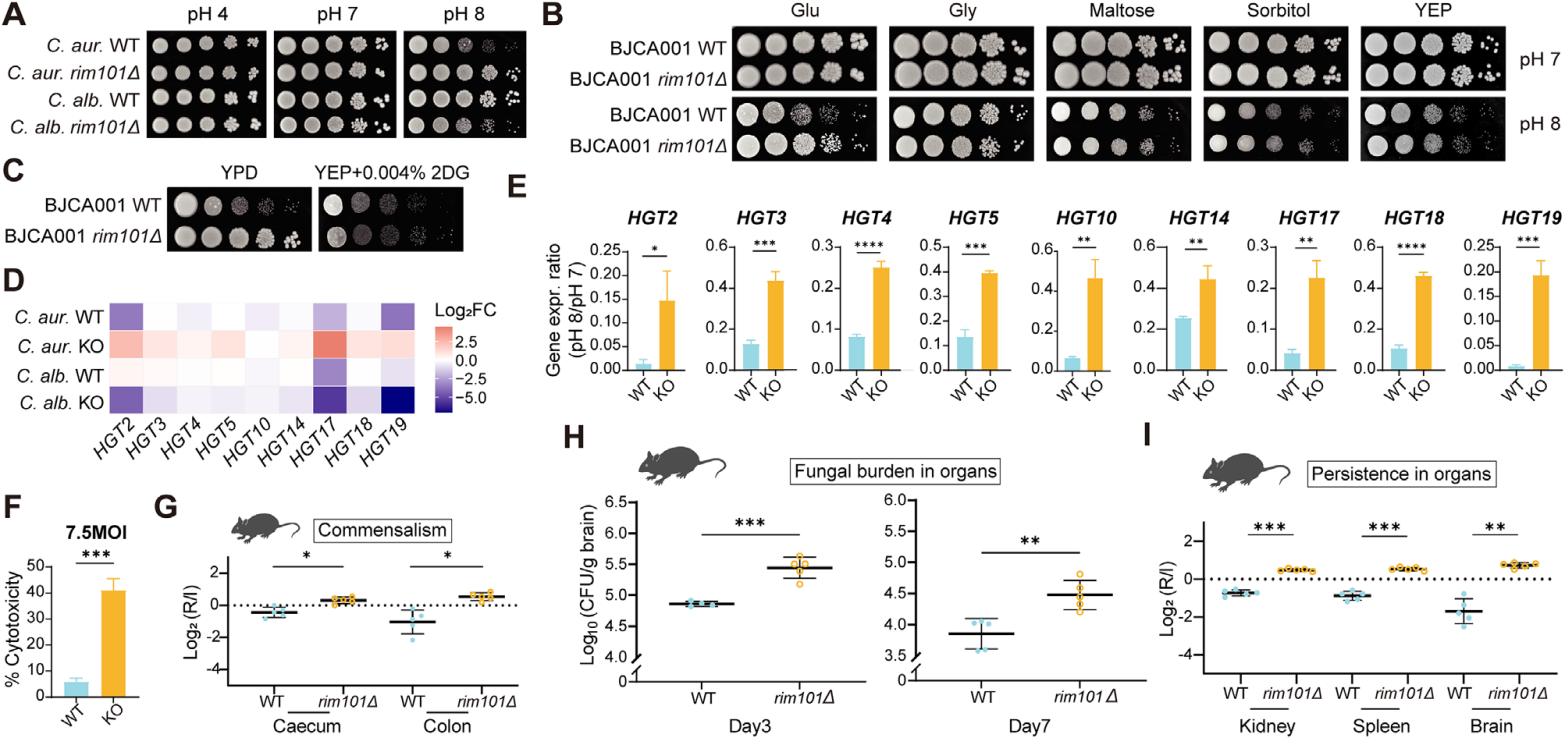
The lack of *RIM101* in *C. auris* promotes both its commensal lifestyle and virulence under the alkaline conditions by relieving glucose repression. (A) The growth phenotypes of the WT and *rim101Δ* mutant strains of *C. auris* and *C. albicans* were observed on a YPD plate with varying pH values after an incubation time of 48 h. (B) The growth phenotypes of the WT and *rim101Δ* mutant strains of *C. auris* were examined after they were cultured for 48 h on alkaline solid media that contained different carbon sources. (C) The growth phenotypes of the WT and *rim101Δ* mutant strains of *C. auris* were examined after they were cultured for 72 h on alkaline solid medium supplemented with 0.004% 2‐deoxyglucose. (D) The expression changes of eight genes associated with glucose uptake and utilization, as revealed by the RNA‐seq analysis, were observed in the WT and *rim101Δ* mutant strains of *C. auris* and *C. albicans* when cultivated on the alkaline solid medium. (E) qRT‐PCR validation of genes in (D). (F) Cytotoxicity assay. LDH concentrations released from J744 cells were detected using CytoTox 96 Non‐Radioactive Cytotoxicity Assay (Promega) after co‐incubation with WT and *rim101Δ* strains, indicating cytotoxicity of the strains. (G) Commensalism experiment. Drinking water containing 4% glucose was given to C57BL/6 mice, after which they were infected by gavage with 1:1 mixture of the *C. auris* WT and *rim101Δ* mutant strains. The abundance of each strain in the inoculum (I) and after recovery from intestinal contents (R) was determined by qPCR. (H) Fungal burden in mouse brains 3 days after infection with *C. auris* WT and *rim101Δ* strains harvested from alkaline solid YPD medium. (I) Competitive differences between WT and *rim101Δ* strains of *C. auris* harvested from alkaline solid YPD medium in different organs of mice 3 days after infection. Data are representative of at least three independent experiments. *p < 0.05, **p < 0.01, ***p < 0.001, ****p < 0.0001 by unpaired Student's *t* test (E,G,H,I).

To systematically validate the functional roles of TFs enabling *C. auris* to adapt to diverse environmental stresses, we generated knockout mutants of candidate regulatory genes identified from our RNA‐seq datasets. Phenotypic analysis revealed no observable alterations in growth or stress tolerance among these mutants, with the notable exception of the *rim101Δ* mutant (Figure S5A,B). Notably, the *C. auris rim101Δ* mutant exhibited robust growth at pH 8.0 when compared to the WT, in sharp contrast to the growth phenotype observed in *C. albicans* (Figure 6A). To validate this phenotypic observation, we first generated multiple independent *rim101* deletion mutants in *C. auris*. Transcriptomic analysis via qRT‐PCR validated the complete absence of *RIM101* gene expression in the mutant background (Figure S5C). Two independently generated *rim101Δ* isolates displayed congruent phenotypic alterations, confirming the specificity of the observed growth phenotype (Figure S5D), confirming the specificity of the observed growth phenotype. Second, geographical isolate analysis across *C. auris* strains from distinct global regions revealed a highly conserved phenotype: all *rim101* deletion mutants exhibited significantly enhanced growth at pH 8.0 compared to their respective WT counterparts (Figure S5D). Again, this phenotypic pattern starkly contrasted with the pH‐dependent growth defects observed in *C. albicans rim101Δ* mutants (Figure 6A). Finally, functional complementation experiments by introducing the *C. auris RIM101* ortholog into *C. albicans rim101* knockout mutant failed to rescue WT growth characteristics under alkaline conditions (Figure S5D). Taken together, these data provide strong evidence for species‐specific evolutionary divergence of *RIM101* gene function, suggesting a specialized regulatory role in *C. auris* adaptation to alkaline environments.

To determine whether the growth advantage conferred by *C. auris rim101* deletion was specific to alkaline stress, we systematically characterized stress response phenotypes across diverse environmental challenges. No significant phenotypic divergence from WT controls was observed (Figure S5E), confirming the specificity of *RIM101*‐mediated resistance to alkaline pH. Intriguingly, substituting glucose in YPD medium with glycerol or other carbon sources completely attenuated the alkaline growth phenotype of *rim101Δ* mutant (Figure 6B). This observation prompted us to hypothesize that *rim101* deletion enhances glucose uptake and utilization under alkaline conditions. To test this, we employed 2‐deoxyglucose (0.004%), a cytotoxic glucose analog transported via glucose transporters but unable to enter glycolysis [60]. The *rim101Δ* mutant displayed pronounced growth inhibition on 2‐deoxyglucose‐containing alkaline medium (Figure 6C), indicating elevated transporter‐mediated cytotoxicity. Furthermore, our qRT‐PCR analysis revealed significant upregulation of all nine members of HGT gene family in *rim101Δ* mutant under alkaline conditions (Figure 6D,E), which is consistent with our hypothesis. Concomitantly, genes encoding hexokinase (*GLK1*, *HXK1*, *HXK2*) involved in the initial phosphorylation step of glycolysis were also robustly upregulated (Figure S5F). These findings collectively demonstrate that *RIM101* deletion coordinately enhances glucose transport and utilization pathways, providing a mechanistic basis for the observed alkaline growth phenotype.

To characterize the metabolic phenotype of *rim101Δ* mutant under alkaline stress (Figure S5G), the fungal cells were cultured on solid YPD medium for 34 hours (h) (Figure S5H). Given the established role of glucose in ATP production, we first measured intracellular ATP accumulation and observed significantly reduced ATP concentrations in *rim101Δ* mutant compared to WT under alkaline conditions (Figure S5I). Flow cytometry analysis revealed that, in contrast to the neutral pH conditions, the WT strain exhibited a pronounced cell cycle arrest at the G2/M phase under alkaline conditions, consistent with growth inhibition observed under alkaline conditions (Figure S5J). This phenotypic convergence suggests that *RIM101*‐mediated growth inhibition under alkaline conditions may involve cell cycle checkpoint control, a process that could be attributed to the dysbiotic glycolysis.

During the commensal‐to‐pathogen transition, *C. albicans* executes transcriptional reprogramming and undergoes morphological differentiation to adapt to distinct host microenvironment [61]. The ability to respond to diverse stresses is therefore critical for the transition between commensal and pathogenic lifestyles. To investigate whether *rim101* deletion impacts virulence potential, *rim101Δ* and WT *C. auris* strains were recovered from solid YPD medium buffered at pH 8.0 and co‐incubated with J744 murine macrophages in DMEM medium for 21 h. The *rim101Δ* mutant elicited significantly higher LDH release compared to WT (Figure 6F and Figure S5K), indicating enhanced cytotoxicity toward host immune cells. Moreover, in our previous work, we identified the critical role of the mannan layer within the *C. auris* cell wall in the innate immune evasion, and this finding is consistent with what has been reported in other studies [62, 63]. Therefore, we used flow cytometry to measure the mannan content in the cell walls of WT and *rim101Δ* strains of *C. auris* under neutral and alkaline conditions, and found that the *rim101Δ* mutant strain had a higher mannan content under alkaline conditions (Figure S5L). This indicates that the *rim101Δ* strain may have a stronger immune escape ability, thereby improving its survival in the host and further enhancing its pathogenicity.

The contribution of *RIM101* to *C. auris* commensalism and virulence was further examined in vivo. Given the progressive alkalization of the gastrointestinal tract from stomach to rectum in both humans and mice, we first evaluated the intestinal colonization capacity of *rim101Δ* mutant relative to WT. Cells collected from alkaline YPD medium were mixed at a ratio of 1:1 and administered intragastrically to C57BL6 mice. Surprisingly, our preliminary analysis of competitive index in cecal and colonic contents indicated that the *rim101Δ* mutant exhibited a marked competitive defect to the WT strain in the gastrointestinal (GI) tract (Figure S5M). This in vivo phenotype is in contrast to our in vitro observations, where deletion of *RIM101* appears to boost pathogenicity through increased cytotoxicity against host cells and more effective immune evasion. Considering the extreme low levels of intestinal glucose, as reported in previous studies [64], and the fact that a commercial kit was unable to detect even trace amounts of glucose in murine intestinal samples (the levels being far below the minimum detectable value of the kit), we hypothesize that the inoculated *C. auris* cells are unlikely to be exposed to a glucose‐abundant intestinal environment within the host. To validate this hypothesis and interrogate the functional significance of glucose availability, we supplemented drinking water with 4% glucose and repeated the colonization experiments. On day 3 post‐inoculation, *rim101Δ* mutant displayed a modest but statistically significant competitive advantage (*p* < 0.05) in cecal and colonic compartments (Figure 6G), as determined by quantitative PCR analysis of strain‐specific primers. These findings suggest that *RIM101*‐mediated glucose metabolism appears to confer a fitness advantage under conditions of glucose supplementation, potentially recapitulating host niche‐specific nutrient availability during colonization.

Given the non‐lethal nature of *C. auris* infection in murine models [6, 62, 65], which precluded survival curve analysis for virulence assessment, we employed alternative strategies focusing on fungal burden quantification and in vivo competitive fitness experiments to characterize the virulence of *rim101Δ* and WT. Organs (kidney, spleen, liver, lung, and brain) were harvested on days 3 and 7 post‐infection to enumerate fungal colony‐forming units (CFU). The *rim101Δ* mutant displayed significantly elevated CFU counts in brain tissue compared to WT at both time points (Figure 6H). For in vivo competitive fitness evaluation, a 1:1 mixture of WT and *rim101Δ* cells (2 × 10^7^ CFU in 200 µL PBS) was administered via tail vein injection. Organs harvested on day 3 revealed that *rim101Δ* mutant outcompeted the WT strain in kidney, spleen, and brain compartments, as determined by strain‐specific qPCR analysis (Figure 6I). The results were further corroborated in our in vivo infection assays using *Galleria mellonella* as the model, where *rim101Δ* mutant‐infected larvae exhibited significantly reduced survival rates compared to the WT strain (Figure S5N), suggestive of a stronger virulent phenotype. Collectively, our in vivo data strongly support that *RIM101* deletion confers a survival advantage to *C. auris* under alkaline conditions, translating to enhanced pathogenicity in mammalian and insect hosts. The observed organ‐specific colonization patterns suggest that *RIM101*‐mediated metabolic reprogramming may facilitate niche adaptation within host tissues characterized by elevated pH and restricted nutrient availability.

## 3 Discussion

The ability to mount adaptive stress responses is critical for fungal commensal‐to‐pathogen transition, making mechanistic investigation pivotal for developing targeted antifungal therapies [66, 67, 68]. *C. auris* is an emerging multidrug‐resistant fungal pathogen that poses a significant threat to human health. Despite sharing conserved drug resistance determinants with *C. albicans* [69, 70], *C. auris* diverges phylogenetically from relatives like *S. cerevisiae* and *C. albicans* (diploid), both belonging to the CTG clade, and maintains a haploid genome [69]. The distinct cell wall mannosylated glycoproteins contribute to its reduced immunogenicity compared to *C. albicans* [62]. Phenotypic profiling reveals that *C. auris* exhibits differential stress resistance compared to *C. albicans*: it tolerates high temperature, salinity, hydrogen peroxide, cell wall perturbation, calcium chloride, and alkaline pH, but is sensitive to superoxide, peroxide, histatin 5, and acidic pH [2, 16, 71, 72, 73, 74, 75]. However, the molecular basis of these stress responses remains poorly understood. Our study provides a pan‐genome structure and comprehensive transcriptomic insights into the stress responses of *C. auris*, and highlights a conserved iron regulon with species‐specific adaptation and functional divergence in alkaline stress‐responsive transcription factor *RIM101* across fungal phyla. These findings advance our understanding of *C. auris* pathogenesis by linking stress adaptation to niche‐specific metabolic reprogramming, with implications for developing antifungal strategies targeting conserved yet functionally divergent pathways.

We characterized the phylogenetic relationships and relative evolutionary divergence of *C. auris* isolates at the genome level, by integrating whole‐genome sequencing (WGS) data from 1,306 isolates spanning 28 countries. Phylogenetic reconstruction identified six major clades (I–VI), with significant phylogeographic intermixing observed across all clades, consistent with previous global surveillance studies [5, 19, 20, 21, 22]. Notably, Clade I contained the largest number of isolates (*n *= 589; including JXCA001 and BJCA001 described previously) and exhibited the most extensive geographical distribution. This contrasts with Clade IV, which demonstrated the strongest phylogeographic structuring [19]. SNP analysis revealed that Clade I harbored a conserved genomic profile, whereas Clade IV displayed the highest genetic diversity, consistent with its evolutionary distinctiveness. In addition, antifungal susceptibility profiling uncovered clade‐specific resistant patterns. Clade I and III harbored significantly higher proportions of fluconazole‐resistant isolates compared to susceptible strains, whereas Clade II and IV showed inverse trends, with predominant susceptible isolates. These patterns are also consistent with previous studies [76, 77]. Functional enrichment analysis identified three novel fluconazole resistance‐associated factors (*HGT7*, *PGA7*, and *FAS2*). The GWAS analysis identified 23 candidate genes associated with the fluconazole resistance of *C. auris* (based on the results from the Clade IV strains), with 10 of them having orthologs in *C. albicans* (including the hexokinase‐encoding gene *HXK2* known to have a major role in the utilization of hexose sugars such as glucose). These candidate genes highlight diverse yet important mechanisms linked to drug resistance, and may serve as putative targets for antifungal therapy development. Previous pan‐genomic studies across *S. cerevisiae*, *C. albicans*, *Cryptococcus neoformans* var. *grubii* and *Aspergillus. fumigatus* reported core gene proportions range from 80%–90% and annotated InterPro domains spanning 68%–79% [78]. In striking contrast, our analysis of *C. auris* isolates uncovered a significantly reduced core genome (58.59%) paired with an expanded InterPro domain repertoire (86.95%), indicative of substantial evolutionary divergence. This compositional shift suggests *C. auris* has undergone accelerated genome evolution characterized by extensive gene family turnover and domain innovation. These findings establish *C. auris* as a globally distributed species with exceptional phylogenetic diversity, which may be distinguished by genomic variability, functional divergence, and phenotypic plasticity. This genome‐wide instability likely contributes to the ecological success of *C. aruis* through rapid adaptation to diverse niches.

To identify key regulatory hubs governing the environmental stress adaptation of *C. auris*, we constructed a co‐regulation network using comparative transcriptomic datasets generated across 32 distinct environmental stress conditions. Employing DEG clustering, WGCNA, and GSEA, we identified co‐expressed genes involved in stress response, partially overlapping those reported in *C. albicans* and *S. cerevisiae*. Moreover, an EGRIN model was applied to identify critical TFs playing essential roles in stress responses, especially those highly conserved TFs such as *RIM101*, *SFU1*, *SEF1*, and *HAP43*. Through gene functional enrichment and expression profiling, we identified *RIM101* as a key regulator for *C. auris* adaptation to alkaline environments and *SFU1*, *SEF1*, and *HAP43* as central regulators for iron regulation. Interestingly, despite being evolutionarily distinct and differing in ploidy (haploid *C. auris* vs. diploid *C. albicans*), these two pathogenic fungi exhibit striking convergence in their iron regulatory mechanisms, as we found that the iron regulatory circuit comprising TFs *SFU1*, *SEF1*, and *HAP43* demonstrates conserved functionality between the two yeasts, with near‐identical operational mechanisms despite their evolutionary divergence. In contrast, the well‐characterized haploid yeast *S. cerevisiae* employs the transcript factor *AFT2* orchestrate iron uptake and utilization, yet its ortholog in *C. auris* exhibits no functional conservation within this regulatory framework. These findings suggest that pathogen *C. auris* has evolved a sophisticated iron‐regulatory network, functionally convergent with *C. albicans* but divergent from *S. cerevisiae*. This regulatory complexity, which was potentially shaped by host‐specific pressures, may enhance its adaptability to the dynamic and hostile host microenvironment, where iron availability fluctuates amid competing stresses.

The discovery that *RIM101* has opposite effects on virulence in *C. auris* and *C. albicans* challenges assumptions about functional conservation in fungal pathogenesis, prompting urgent mechanistic and evolutionary studies. *C. albicans*, the most common opportunistic fungus in humans, rapidly adapts to the host via yeast‐to‐hypha transition, enabling its shift from commensal to pathogen [79, 80]. This transition is influenced by the interaction among the environment, the host, and the pathogen. In contrast, *C. auris*, an emerging multidrug‐resistant pathogen, may have evolved in response to environmental stresses and host immune defense strategies, though its underlying mechanisms remain unclear. In addition, searching the orthologs operating in the conserved *RIM* pathway, a common pH response pathway critical for fungal adaptation to alkaline pH in *S. cerevisiae*, as well as the pathogenic fungi *C. albicans* and *C. neoformans*, yields a very interesting phenomenon. The genes involved in *RIM* pathways, including those required for *RIM101* activation, such as *RIM9*, *RIM21*, *RIM8*, *RIM20*, and *RIM13*, as well as *RIM101* itself or downstream targets such as *HSP90*, *IPT1*, *NRG1*, and *VPH1*, share low sequence identities between *C. auris* and *C. albicans*. For example, *NRG1* is the well‐characterized *RIM101* target, and only 25.46% sequence identity was observed in the two species. We speculate that the variations in the *RIM* pathway and its downstream gene sequences have led to the differences in gene regulation of *C. auris* under alkaline environmental stresses, ultimately resulting in the growth phenotypic differences. This difference in regulatory pathways may have arisen from the adaptive evolution of *C. auris* as a “super fungus,” allowing it to withstand higher environmental stresses during evolution. More importantly, our experiments further demonstrated that the stronger virulence and symbiotic ability of *C. auris* in the absence of *RIM101* may mainly depend on its stress resistance mechanism in response to harsh environments. Depletion of *rim101* in *C. auris* leads to growth restriction under alkaline conditions, triggering the activation of stress resistance gene expression. This response primarily involves upregulation of the glycolysis pathway to enhance glucose absorption and utilization, thereby improving its growth further improving virulence. The stress resistance is a key characteristic of this superbug, making it extremely difficult to eliminate from medical environments using common disinfection methods. Therefore, further investigation on how *C. auris* activates the expression of its stress resistance genes as well as exploration of related pathways, will be crucial in developing effective strategies to combat *C. auris* infections in healthcare settings.

A striking glucose uptake divergence was observed between *C. auris* and *C. albicans rim101Δ* mutants under alkaline conditions: *C. auris rim101Δ* mutant displayed a significant increase in glucose uptake via upregulated hexose transporters (HGT family) and kinases (*HXK1/HXK2*, *GLK1*), whereas *C. albicans rim101Δ* exhibited reduction in glucose utilization, when cells were grown under alkaline conditions. This phenotypic contrast maps to differential regulation of glucose metabolism pathways, contradicting the classical glucose repression effect in *S. cerevisiae*, which was regulated by *SNF3*/*RGT2* and *SNF1* (or their orthologous genes in *C. auris*, that is, *HGT2* etc.) pathways [27, 81, 82, 83, 84, 85]. In *S. cerevisiae*, glucose limitation triggers downregulation of the *SNF3*/*RGT2* pathway and upregulation of *SNF1* expression, relieving the repression of *MIG1*/*MIG2* and activating the expression of downstream target genes such as the HGT family. In addition, it was previously reported that under basal conditions, genes related to the glycolytic pathway were downregulated while those associated with the TCA cycle were upregulated in *C. auris* when compared to *C. albicans* [72, 86]. However, our study provides the first evidence that *C. auris RIM101* modulates glucose transporter gene expression under alkaline stress by relieving glucose repression, a regulatory mechanism distinct from its orthologue in *C. albicans*. This functional divergence was further corroborated by phenotypic assays demonstrating enhanced virulence and competitive fitness in *rim101Δ* mutant under alkaline conditions. Our observations argue that *RIM101* may act as a lineage‐specific metabolic rheostat regulating stress adaptation in *C. auris*.

In summary, our study presents the first comprehensive characterization of core regulatory modules underpinning *C. auris* adaptation to dynamic host microenvironments. Through integrative genomics and functional validation, we identified conserved stress‐response networks rewired by species‐specific TFs, providing mechanistic insights into the ecological versatility of *C. auris*. These insights establish a framework for developing precision medicine strategies targeting evolutionarily divergent transcriptional regulators, which may effectively tackle drug resistance issues in fungal infections.

## 4 Methods

### 4.1 Data Collection and SNP Identification Workflow

The whole‐genome sequence data of 1,306 *C. auris* isolates comprised both public data on NCBI and our own data. They come from 46 BioProjects and are related to 35 studies [5, 15, 20, 21, 69, 71, 77, 87, 88, 89, 90, 91, 92, 93, 94, 95, 96, 97, 98, 99, 100, 101, 102, 103, 104, 105, 106, 107, 108, 109, 110, 111, 112, 113, 114, 115, 116]. Details of strains, including geographic location, sample type, isolation source, and so on are available in Table S1.

Raw sequence data were quality‐checked using FastQC v0.11.9 (https://www.bioinformatics.babraham.ac.uk/projects/fastqc/) and trimmed/filtered with PRINSEQ v0.20.4 [117], to remove low‐quality bases and adapter contaminants. High‐quality reads were aligned to the *C. auris* assembly strain B8441 (GCA_002759435.2) using BWA mem v0.7.17 [118]. SNP calling was performed with GATK v4.1.9 [119]. Only those variants that met the filtering criteria (QD ≥ 2.0 || FS ≤ 60.0 || MQ ≥ 40.0) were retained. Genotypes were filtered using filterGatkGenotypes.py with parameter “‐min_GQ 50 ‐min_percent_alt_in_AD 0.8 ‐min_total_DP 10” (https://github.com/broadinstitute/broad‐fungalgroup/blob/master/scripts/SNPs/filterGatkGenotypes.py).

### 4.2 Phylogenetic Analyses

A subset of high‐quality SNPs (*n* = 506,345) passing variant and genotype filters was selected for phylogenetic reconstruction. Phylogenetic trees were inferred using IQ‐TREE v2.1.4 [120] under the GTR model based on 1,000 replicates. Clade‐specific trees were visualized and annotated using iTOL [121].

### 4.3 Pan‐Genome Construction and Annotation

The adapters and low‐quality sequences of the raw reads of all strains were trimmed by Trimmomatic [122] v0.39 with parameters “‐length 20 ‐stringency 3”. The final high‐quality cleaned reads from 1,306 strains were *de novo* assembled respectively using SPAdes v3.15.3 [123] with default parameters.

To identify possible redundancies among assembled contigs that were already present in the reference genome, the assembled contigs were concatenated and were aligned to the reference genome using NUCmer v.4.0.0rc1 [124] with default parameters “‐l 20 ‐c 65” and the alignments with length ≥ 300 bp and identity of greater than 90% sequencing region were thought as redundant region to be removed. The rest region longer than 300 bp and unaligned contigs were kept as “novel” sequences. The unaligned contigs and unaligned sequences (>300 bp) were searched against the GenBank NT database by blastn [125]. Sequences with best hits from outside the *Candida*, ascomycetes, basidiomycetes, budding yeasts, and unclassified sequences were thought of as potential contaminations and removed.

To further remove redundancies in the cleaned non‐reference sequences, all‐versus‐all alignment was performed using CD‐HIT [126], nucmer, and blastn. The same process was performed iteratively until no redundancies were left. In all of the above filtering processes, the sequence identity threshold was set to 90%, and the sequence length threshold was set to >300 bp. The final non‐redundant non‐reference sequences and the reference genome [97] were concatenated to construct the *C. auris* pan‐genome.

The novel genome was predicted and annotated by Funannotate pipeline v1.8.9 [127]. To build a custom repeat library for masking repeat sequences, RepeatModeler v1.0.11 (http://www.repeatmasker.org/RepeatModeler/) was used to perform de novo prediction of repeat sequences; RepeatMasker v4.1.2‐p1 (http://www.repeatmasker.org) with parameter “‐species Candida”) was used to identify repeat sequences based on known repeat databases (Dfam and RepBase). The repeat sequences obtained from these two approaches were subsequently combined to generate a repeat library, which was used by Funannotate mask module.

*De novo* gene models for the repeat‐masked non‐reference genome sequences were predicted using the Funannotate predict module. In this approach, GeneMark‐ES v4.68_lic [128] and AUGUSTUS v3.3.3 [129] were used to predict genes, and DIAMOND v2.0.13 [130] was used to generate evidence‐based gene models by aligning the contig sequences with the protein sequence database (UniProtKB). EVidenceModeler v1.1.1 [131] with default weighting was applied to select the consensus models.

The functional annotation of protein was predicted using the Funannotate annotate module, which searched for and identified proteins through several databases, including Pfam v34.0 [132], BUSCO v2.0 [133], EggNOG‐mapper v2.1.1 [134], MEROPS v12.0 [135], and CAZyme v9.0 [136]. SignalP [137] was used to predict secretome, while Phobius [138] was used to predict both secreted and transmembrane proteins. The tRNA genes were identified by using tRNAscan‐SE v.2.0.9 [139]. The secondary metabolite was predicted using antiSMASH v6.0.1 [140].

### 4.4 Analysis of Gene Presence and Absence

The pair‐end raw reads from 1,306 *C. auris* strain were aligned to the pan‐genome using BWA‐MEM2 [141]. The coverage of each gene and CDS regions was calculated by bedtools [142]. Gene coverage <80% or CDS coverage <95% was classified as absent, otherwise, genes were considered present. Functional enrichment analysis among gene sets was performed in *R*, with Fisher's exact test and Bonferroni's correction of *p‐*values.

### 4.5 Identification of GWAS Signals Associated With Drug Resistance

We first performed quality control filtering to remove certain sites: (1) sites with sequencing depth lower than 5, (2) sites with genotype quality below 20, (3) sites missing in more than 10% of strains within Clade IV, (4) sites with minor allele frequency (MAF) below 5% or above 95%, and (5) sites in linkage disequilibrium (LD). LD filtering was conducted using plink2 v2.00a5.12LM [143] with the parameters “‐allow‐extra‐chr ‐indep‐pairwise 50 5 0.5” where 50 represents the sliding window size, 5 is the step size, and 0.5 is the LD threshold (only SNPs with *R* ^2^ < 0.5 are retained). Next, we performed PCA using plink2 to assess the population structure within Clade IV. Finally, the principal components obtained from PCA are included as covariates in a logistic regression model to identify drug resistance‐associated mutations. The results were adjusted using the Bonferroni correction for *p* values. We identified significant SNP loci and performed functional annotation with SnpEff [144] to detect key variants significantly associated with fluconazole resistance.

### 4.6 DEG and GSEA

The DESeq2 [145] tool was employed to conduct differential analysis on each batch of samples, applying a fold change threshold of 2, False Discovery Rate (FDR) correction for *p* values, and a significance threshold of 0.05 to identify differentially expressed genes across all conditions. To mitigate batch effects, control groups were meticulously designated for each experimental batch, ensuring the absence of confounding batch‐related variations in gene differential expression analysis. Addressing varying cardinalities of gene expression values, Pearson correlation distance was applied to circumvent dimension and outlier influences. Ultimately, Ward's clustering criterion was implemented to minimize intra‐class differences and accentuate inter‐class disparities in the clustering outcomes.

The raw count matrices were normalized through the Variance‐Stabilizing Transformation (VST) method. Limma [146] was applied to regress most batch effects. PCA of preprocessed data confirmed effective batch removal, with ANOVA on the first principal component yielding a highly significant *p*‐value.

Functional enrichment analysis of KEGG and GO pathways was performed using Over‐Representation Analysis (ORA) and GSEA methods. Benjamini–Hochberg correction and a threshold of 0.05 were applied to *p*‐values for selection of significant pathways.

### 4.7 WGCNA Analysis

WGCNA analysis was conducted on the VST normalized and batch corrected data. The scale‐free topology criterion was applied to determine the lowest soft threshold power guaranteeing a scale‐free topology fit (*R* ^2^ >0.9). The co‐expression modules were constructed using the automatic network construction function “blockwiseModules” with the following settings: power = 6; minModuleSize = 30; reassignThreshold = 0; mergeCutHeight = 0.25; and TOMType = unsigned. All other parameters were set to default values. All genes were clustered into 10 modules, and generated a Topological Overlap Matrix (TOM) that used for constructing the gene co‐expression network. Modules with *p*‐value <0.0001 and an absolute correlation coefficient >0.5 were considered as significantly associated with traits (i.e., environmental stresses). In each module, we regarded each gene as a source and selected two genes with the highest degree of correlation as their regulated targets. Then we chose target genes whose number of sources was more than zero to build regulatory networks (visualized with Cytoscape [147]). Finally, we marked a number of hub‐genes (core genes) which inferred from each module (according to high module membership (kME) values).

### 4.8 LncRNA Annotation

The alignment of strand‐specific data to the genomic landscape was executed using the STAR v2.6a [148]. For each sample we performed genome‐guided transcriptome assembly using Stringtie v1.3.3b [149]. For each species, we merged all assembly .gtf files produced by Stringtie from all samples using option “‐g 50” which resulted in a unified transcriptome. These transcriptome annotations were compared with the original genome annotations of each species using gff‐compare v0.11.2 [150] to identify novel transcripts. We selected novel intergenic (“i”) and antisense (“a”) transcripts (corresponding to “u” and “x” class‐codes of gffcompare output, respectively) longer than 200 bp. When several isoforms were present, we kept only the longest one using CGAT gtf2gtf v.0.3.2 [151] software. The identification of transcripts as non‐coding protein sequences was performed through the utilization of three distinct software tools: PLEK [152], CNCI [153], and CPC2 [154]. Transcripts concurrently identified as non‐coding by all three aforementioned software tools were retained and classified as lncRNAs.

### 4.9 Cross‐Transcriptome Comparison of GSEA Outcome

Public transcriptome data under six stress conditions (pH 8 [155], H_2_O_2_ [155], high copper [156, 157], low iron [44], MMS [35], and hypoxia [158]) were collected from species including *C. auris*, *C. albicans*, *C. glabrata*, *C. parapsilosis*, and *S. cerevisiae*. Under each condition, the R package clusterProfiler [159] was applied to calculate significant enrichment GO terms of each species and the intersection was taken. Normalized enrichment score (NES) was ranked to shortlist the top 10 positively and negatively enriched terms, which were then visualized via a bar plot.

### 4.10 cMonkey2

We used cMonkey2 [52] to identify co‐regulated gene modules and their associated conditions within 138 *C. auris* transcriptomes. During the gene module detection process, cMonkey2 carried out functional annotation using our in‐house database. To optimize the co‐regulated gene modules, we ran 2,000 iterations of cMonkey2. In each iteration, the software refined the gene modules by evaluating and, if needed, modifying the memberships of both conditions and genes. Each gene module covered 1–48 genes. To assess the quality of the identified modules, we calculated the residual scores, which served as indicators of the coherence of gene co‐expression patterns [160]. Modules with lower residual scores were considered to be of higher quality. Based on an empirical approach established in a previous study of *Mycobacterium tuberculosis* [161], we set a residual cutoff of 0.55 to select functionally significant modules.

### 4.11 Animal Manipulation

Female C57BL/6 mice (6–8 weeks old, 18–20 g body weight) were purchased from Beijing Vitong Lever Laboratory Animal Technology Co., Ltd. (Beijing, China). Mice were routinely housed in a pathogen‐free animal facility at 21°C, 50%–70% relative humidity, and under a constant 12‐h light/dark cycle, with ad libitum access to autoclaved food and filtered water. All procedures were performed in accordance with protocols approved by the Institutional Animal Care and Use Committee (IACUC) at Shanghai Institute of Immunity and Infection, Chinese Academy of Sciences. *G. mellonella* larvae were purchased from Tianjin Huiyude Company. Larvae were selected to ensure high vitality (uniform size and motility), and then assigned to experimental groups using a random number generator to ensure equivalent body weight distributions. Infected larvae were incubated at 30°C in 90 mm Petri dishes containing sterile filter paper to maintain humidity.

### 4.12 Preparation of Fungal Cells

All strains used in the experiments are listed in Table S11. All in vitro and in vivo studies used the *C. auris* strain BJCA001 (the first clinical isolate of *C. auris* in China). In addition, six clinical isolates from different geographical areas were used to verify the conservation of alkaline tolerance pathways induced by *rim101*. These strains include one from China (Cau‐C #2, Clade I), two from Japan (Cau‐J #1–2, Clade II), two from India (Cau‐I #1‐2, Clade I), and one from Korea (Cau‐K, Clade II). Strains were obtained from glycerol stocks stored at −80°C, plated on YPD (2% Bacto‐peptone, 1% yeast extract, 2% glucose) and grown routinely at 30°C. Fungal samples used in in vitro and in vivo experiments were cultured in liquid and solid media. For liquid culture conditions, cell suspensions were prepared by transferring the fungal cells to YPD broth and incubating with shaking at 30°C for the indicated time. Normally, cells were grown exponentially to an OD_600_ of 0.6–0.8 and washed three times with PBS prior to cell culturing or animal administration. For solid culture conditions, 2 × 10^5^ *C. auris* cells cultured overnight were spread onto 10 cm YPD agar plates and incubated at 30°C for 34 h. Cells were collected by scraping and washed three times with PBS, and adjusted to the desired concentration in PBS for murine infection or macrophage co‐culture experiments.

### 4.13 Medium

Liquid YPD broth was normally used for fungal cultivation. Any specific culture media are detailed in Table S7. The “iron‐limited” medium was YPD plus 500 µM biphenanthroline disulfonic acid (BPS). For the preparation of YPD solid medium with different pH values in the experiment, 150 mM HEPES was added to 2xYPD liquid medium, then NaOH and HCl were used to adjust to the target pH value, a 22 µm filter was used to filter and sterilize, and finally the same volume of 4% agar and appropriate carbon sources were added.

### 4.14 Reagents

All reagents used in this study are listed in Table S13.

### 4.15 RNA Extraction and Sequencing

Transcriptional profiles of *C. auris* under diverse environmental stresses were systematically characterized with culture conditions detailed in Table S7. All experimental and control groups included three biological replicates. Fungal samples were processed in multiple batches, each accompanied by a contemporaneous control group to account for batch‐to‐batch variability. Cultures were agitated at 60 rpm in an orbital shaker. Standard incubation temperature was 30°C unless otherwise specified. Overnight pre‐cultures were adjusted to OD_600_ = 0.8–1.0 prior to experimental use via optical density measurement. *C. auris* cultures were adjusted to OD_600_ = 0.1–0.2 and incubated for the indicated time periods. Cells were harvested by centrifugation (4,000 rpm, 5 min) for liquid cultures, or washed with PBS for solid cultures. Pellets were snap‐frozen in liquid nitrogen and stored at −80°C until processing. Total RNA was extracted using the *Candida* total RNA extraction method and RNA integrity was evaluated via 1% agarose gel electrophoresis, and concentration was quantified using a Qubit fluorometer. mRNA was prepared from total RNA samples, and RNA quality was assessed and sequenced on an Illumina NovaSeq 6000 platform of Beijing Novogene Bioinformatics Technology Co., Ltd.

### 4.16 Strain Construction

Construction of *C. auris* mutants was performed using a protocol developed for *C. albicans* [162]. The gene knockout approach, based on the principle of homologous recombination, comprises three major steps. Step 1: Fusion PCR was used to obtain the homologous recombination insert fragment. The upstream and downstream flanking regions of the selected genes, each approximately 1,000 bp in length, were amplified using appropriate primers. Step 2: The dominant marker NAT was PCR amplified from plasmid pSFS2A, and subsequently, electroporation was carried out using the GenPulser Xcell TM electroporation system (BioRad) according to the manufacturer's instructions. This process replaced the target gene with the resistance gene. Step 3: Colony PCR was used to screen for positive clones, thereby obtaining the knockout strains. Primers used for PCR amplification are listed in Table S12 and plasmids used for gene deletion are listed in Table S11.

### 4.17 qRT‐PCR Analysis

RNA samples were prepared as described above. cDNA was prepared from DNAse‐treated total RNA samples (1 µg) using PrimeScript RT reagent Kit with gDNA Eraser (RR047A) (TaKaRa). qRT‐PCR was performed on ABI QuantStudio 6 flex (Applied Biosystems). TB Green Premix Ex Taq II (Tli RNase H Plus) (TaKaRa) was used as the reaction mixture, and cDNA was diluted 50 times as the template. The reaction system and cycling conditions were carried out according to the TB Green Premix Ex Taq II operation manual. All experiments were performed using three independent cultures, and each cDNA sample was analyzed in triplicate using each primer pair. Melting curve analysis was performed at the end of the reaction to confirm the presence of a single PCR product. Data were normalized to amplified actin cDNA in each set of PCR experiments. Relative expression levels were determined by the 2^–Δ^ *^C^* ^t^ method. Statistical analysis was performed by two‐tailed *t‐*test. Primers used in these analyses are listed in Table S12.

### 4.18 In Vitro Growth Assay

A single *C. auris* colony was inoculated into 5 mL YPD broth and incubated at 30°C overnight. Optical density at 600 nm (OD_600_) was measured using a spectrophotometer, and cultures were adjusted to OD_600 _= 1.0 with sterile ddH_2_O. Serial 10‐fold dilutions were prepared in sterile PBS and 4 µL aliquots of each dilution were spotted onto YPD agar plates. Plates were incubated and imaged to document colony growth dynamics.

### 4.19 Assay for Intracellular ATP Levels

The ATP detection kit (S0026; Beyotime) were used to measure intracellular ATP of *C. auris* cells. Fungal samples were harvested from solid medium, and cell density was adjusted to OD_600_ = 20 in a total volume of 1 mL. Cell pellets were collected by centrifugation at 10,000 g for 3 min (4°C) and resuspended in buffer containing 100 mM Tris‐HCl (pH = 8.0), 25 mM ammonium acetate, and 4 mM EDTA. The cell suspension was transferred to precooled tubes containing glass beads and disrupted (Bio‐Spec; 5 × 1 min, 6 M s^−1^, 5 min on ice between pulses). After centrifugation, the precipitated proteins were collected by centrifugation at maximum speed (14,000 g) for 10 min (4°C). After discarding the supernatants, the pellets were mixed with 30 µL of 0.2 M NaOH. The neutralized pellet was further dissolved in 370 µL RIPA buffer (pH = 8.0) to resolubilize the protein. After clarification of the lysate at 12,000 rpm for 10 min (4°C), the supernatant was diluted and analyzed for protein concentration using the bicinchoninic acid (BCA; Sigma) assay. The ATP in the supernatant was measured according to the instructions. ATP levels were normalized to the total protein concentration and reported as mean values from three biological replicates. The experiment was repeated twice to ensure the reliability of the results.

### 4.20 Cell Cycle Analysis

*C. auris* cells harvested from solid YPD medium were prepared as previously described and adjusted to 1 × 10^8^ cells mL^−1^ in sterile PBS. Cells were fixed, washed, and resuspended in sodium citrate buffer (pH = 5.5). Cells were stained with propidium iodide (PI; Sigma) and samples were analyzed on a BD FACSCanto II flow cytometer (BD Biosciences) according to the manufactural directions. Data were analyzed with FlowJo software by gating on single‐cell populations and quantifying DNA content distributions in G1/G2 phases.

### 4.21 Flow Cytometric Analysis of Mannan Content in Fungal Cell Wall

To quantitatively measure the content of mannan in the cell wall, we used a previously described method [62]. Yeast cells of *C. auris* (1 × 10^7^) were prepared using the solid medium culture method, washed three times with PBS, and fixed with 4% paraformaldehyde for 1 h at room temperature or overnight at 4°C. For mannan labeling, fixed cells were washed with PBS and stained with concanavalin A (ConA; 50 µg mL^−1^) for 1 h at 37°C in the dark. Data was acquired using a BD LSR Fortessa flow cytometer (BD Biosciences). Data was analyzed using FlowJo software (Treestar, Ashland, OR, USA). All presented data was representative of three biological replicates.

### 4.22 LDH‐Based Cytotoxicity Assay

The J744 cells were cultured in DMEM (Dulbecco's modified Eagle medium) medium containing 10% FBS and 1% penicillin, and seeded in a 96‐well plate at a density of 1×10^5^ cells well^−1^ for 14 h. All cells were maintained at 37°C in a humidified atmosphere containing 5% CO2. The preparation of fungal samples harvested from solid medium was conducted as described above and the cell density was adjusted to 1×10^8^ per ml with PBS before co‐culture with J744 murine macrophages, based on the multiplicity of infection (MOI) of 5, 7.5, or 10. The samples were cultured for 14 h and replaced with new DMEM medium. After incubation for 21 h at 37°C in the incubator containing 5% CO_2_, the relative concentration of LDH in supernatants was detected using CytoTox 96 Non‐Radioactive Cytotoxicity Assay (Promega). Three biological replicates were applied for each strain, and blank and positive controls were prepared according to the kit instructions.

### 4.23 *Drosophila* Infection Assays

Fungal inoculum was prepared from solid YPD cultures as previously described. Cells were harvested, washed twice with sterile PBS, and adjusted to 2×10^7^ CFU mL^−1^. Using a Nanoject II (Drummond) microinjection system, 50.6 nL aliquots were microinjected into the anterior thoracic hemocoel of Ore‐R *D. melanogaster* flies. Infected flies were maintained at 29°C under a 12‐h light/dark cycle with ad libitum access to standard *Drosophila* medium. Mortality was recorded daily, and surviving flies were transferred to fresh vials. Survival curves were generated using the Kaplan–Meier method and analyzed via the log‐rank test.

### 4.24 *Galleria mellonella* Infection Assays

*G. mellonella* larvae were selected for uniform motility and randomly assigned to experimental groups using a random number generator to ensure equivalent total distributions. Fungal cells harvested from solid YPD medium were prepared as previously described, washed twice with sterile PBS, and adjusted to 1 × 10^8^ CFU mL^−1^. A 50 µL microsyringe with 0.26 mm needle was used to inject10 µL of fungal suspension into the second left proleg of each larva. PBS‐injected larvae served as negative controls. Infected larvae were incubated at 30°C in 90 mm Petri dishes containing sterile filter paper. Survival was recorded daily for 7 days. Kaplan–Meier survival curves were generated and analyzed using the log‐rank test.

### 4.25 Murine Model of *Candida* Gastrointestinal Colonization

Six‐week‐old female C57BL/6 mice were administered piperacillin‐tazobactam (PTZ; 8:1 ratio, 320 mg kg^−1^) via subcutaneous injection daily starting on Day 3 to deplete intestinal microbiota. Fecal samples collected on Day 5 were homogenized in 1 mL PBS and plated onto Sabouraud agar containing 50 µg mL^−1^ ampicillin and 15 µg mL^−1^ gentamycin to confirm *C. auris* absence. Fungal cells harvested from solid YPD medium were adjusted to 1 × 10^9^ CFU mL^−1^ in PBS. WT and mutant strains were mixed at a 1:1 ratio, with 200 µL aliquots stored at −80°C as input controls. Mice received 100 µL of the strain mixture via oral gavage on Day 6. Cecal and colonic contents were collected on Day 9 post‐infection into sterile 1.5 mL Eppendorf tubes. Samples were homogenized in 1 mL sterile water via vortexing (maximum speed, 10 min), then 200 µL undiluted homogenate was plated onto selective Sabouraud agar. Colonies were scraped from plates using a sterile cell spreader and resuspended in PBS. Fifty microliters of each suspension were centrifuged (6,000 × *g*, 2 min), supernatants discarded, and pellets snap‐frozen in liquid nitrogen prior to −80°C storage. Genomic DNA was extracted using the standard protocol, and qPCR primers were designed based on the corresponding gene ORF fragment and NAT resistance fragment to identify the relative abundance of WT and mutant strains. Relative abundance of strains was calculated using the ΔΔ*C*t method, normalized to input controls.

### 4.26 Murine Model of Systemic Candidiasis

Female C57BL/6 mice (6–8 weeks old, 18–20 g body weight) were inoculated via tail vein injection with fungal cells prepared from solid YPD cultures. Cells were adjusted to 1 × 10^8^ CFU mL^−1^ in sterile PBS, and 200 µL aliquots were administered (*n* = 5 per group). Infected mice were euthanized on days 3 and 7 post‐infection. Organs (kidneys, spleens, livers, lungs, and brains) were aseptically harvested, weighed, homogenized, serially diluted, and plated on YPD agar. Fungal CFUs were enumerated after 48 h incubation at 30°C, with fungal burden expressed as log_10_ CFU g^−1^ tissue. A 1:1 mixture of WT and knockout mutant strains (1 × 10^8^ CFU for each strain) was prepared, with 200 µL stored as an input control. Mice received 200 µL of the mixture via tail vein injection. The relative abundance of WT and mutant strains was determined as previously described on day 3 post‐infection.

### 4.27 Statistical Analysis

Statistical parameters, including exact values of *n* and statistical significance, are reported in figures and figure legends. Statistical tests were performed using GraphPad PRISM software 8.0.2 (GraphPad Software, Boston, Massachusetts, USA, www.graphpad.com). Specific statistical tests are shown in the figure legends.

## Author Contributions

D.‐S.Z., N.‐N.L., and C.‐B.C. conceived and designed the study. C.‐Y.X., X.‐Q.C., X.‐H.H., Y.‐Y.W., W.‐W.X., Y.Z., L.‐Y.Z., K.‐L.L., Y.‐R.T., J.‐H.L., S.‐C.X., and L.W. performed the experiments. W.‐X.X., Z.‐Y, Y.‐S.Y., X.‐Q.C., O.‐Y.M., Y.‐M.D., G.‐S.C., Y.‐P.Z., Z.‐L.Z., M.G., X.‐T.H., Z.‐Y.H., L.Z., and L.‐B.Z. carried out the data analysis. N.‐N.L., L.‐F. H, C.‐B.C., P.H., and X.‐Q.Z. provided animals, facilities, and technical guidance. C.‐Y.X., W.‐X.X., Y.‐S.Y., X.‐Q.C., and O.‐Y.M. drafted the manuscript. D.‐S.Z., N.‐N.L., C.‐B.C., P.H., X.‐Q.Z., C.‐Y.X., and W.‐X.X. revised the manuscript. All authors discussed the experiments and results and approved the manuscript. All authors have read the final manuscript and approved it for publication.

## Conflicts of Interest

The authors declare no conflicts of interest.

## Ethics Statement

All animal experiments were performed in compliance with the Regulations for the Care and Use of Laboratory Animals issued by the Ministry of Science and Technology of the People's Republic of China, which enforces the ethical use of animals. The protocol was approved by Institutional Animal Care and Use Committee (IACUC) at Shanghai Institute of Immunity and Infection, Chinese Academy of Sciences (Permit Number: A2022029).

## Supporting information




**Supporting File 1**: exp270091‐sup‐0001‐SuppMat.docx

**Supporting File 2**: exp270091‐sup‐0002‐SuppTables.xlsx

## Data Availability

Raw DNA‐seq data have been deposited in NCBI database with BioProject accession number PRJNA1067209. Raw RNA‐seq data are available in GEO database, under the series number GSE255158. Source codes are available upon reasonable request from the corresponding author.
