# Supplementary material for: Global Stress Responses Identify the Functionally Divergent Regulators Required for Candida auris Commensalism and Pathogenicity
Source: Exploration (Beijing). 2025 Nov 11;5(6):20240482. doi: 10.1002/EXP.20240482 (PMC12752650; doi:10.1002/EXP.20240482)
Supplement: Supplementary file 1 — Supporting File 1: exp270091‐sup‐0001‐SuppMat.docx [file EXP2-5-20240482-s002.docx]

**Supporting information**

**Global stress responses identify the functionally divergent regulators required for *Candida auris* commensalism and pathogenicity**

Chaoyue Xu^1,2#^, Wanxing Xu^3#^, Yushun Yuan^4#^, Xiaoqing Chen^1#^, Ouyang Mo^1#^, Zhe Yin^5^, Xinhua Huang^1^, Yuanyuan Wang^1^, Lingfei Hu^5^, Wenwen Xue^2^, Yun Zou^1^, Luyao Zhang^1^, Kunlin Li^1^, Yueru Tian^6^, Jihong Liu^1^, Sichu Xiong^1^, Lei Wu^1^, Yanmei Dong^7^, Guangsheng Chen^8^, Yuping Zhang^9^, Zili Zhou^1^, Ming Guan^6^, Xiaotian Huang^9^, Zhiyi He^8^, Lin Zhong^10^, Lingbing Zeng^11^, Pei Hao^1^*, Xiaoqi Zheng^3^*, Changbin Chen^1,2^*, Ning-Ning Liu^3^*, Dongsheng Zhou^5^*

^#^ These authors contributed equally.

* Corresponding authors: Dongsheng Zhou, dongshengzhou1977@gmail.com; Ning-Ning Liu, fenghu704@163.com; Changbin Chen, cbchen@ips.ac.cn; Xiaoqi Zheng, xqzheng@shsmu.edu.cn; Pei Hao, phao@siii.cas.cn.

**Contents:**

Supporting Figures S1 to S5

Legends for Supporting Tables S1 to S13

**Supporting Figures**


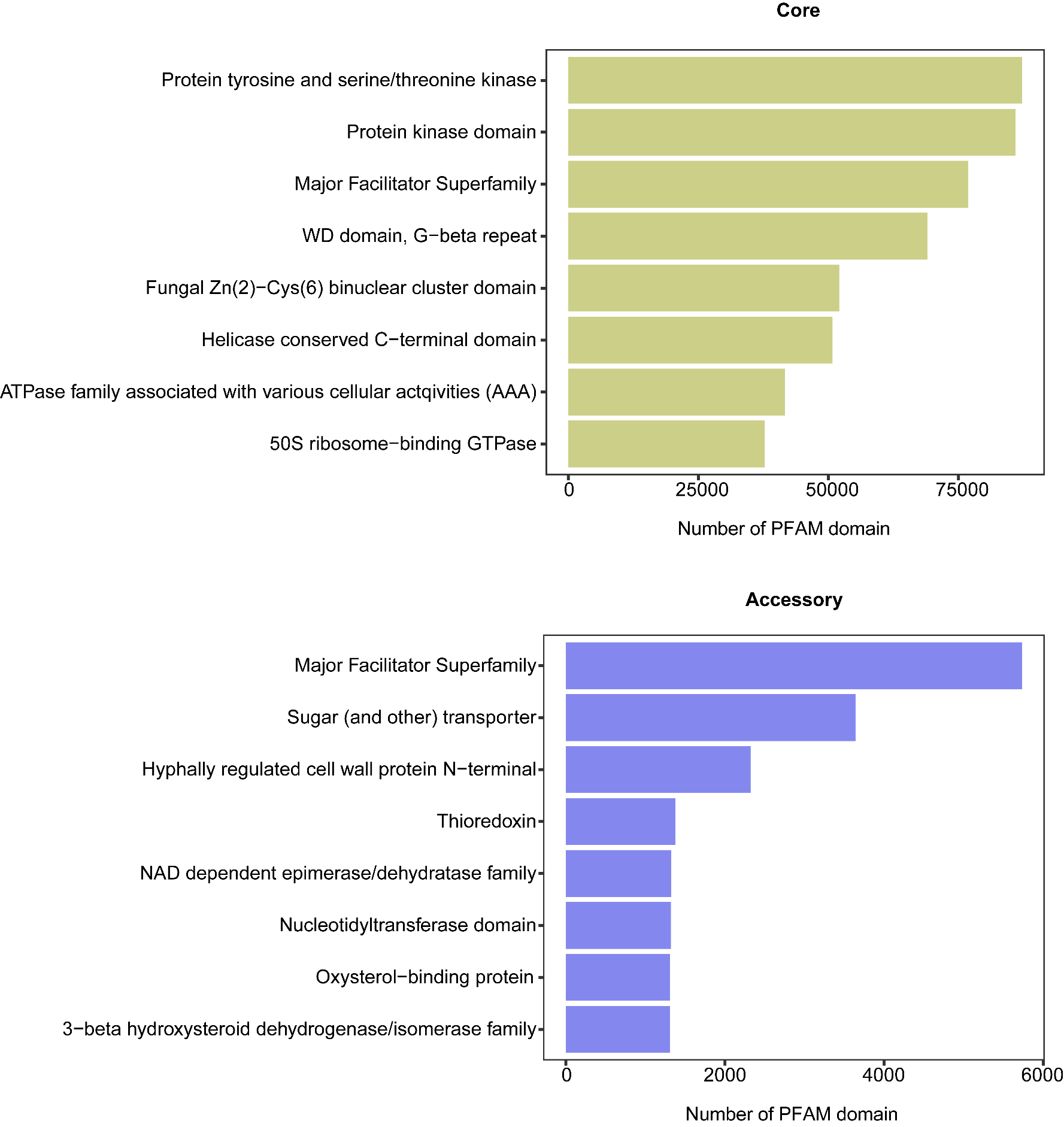


**Figure S1. The top Pfam domains in the core and accessory pan-genomes of *C. auris*.**

The histogram presents the most commonly encountered Pfam domains identified within the core (displayed at the top) and accessory genomes (shown at the bottom). The numerical values denote the total number of proteins encompassing these domains across all 1,306 genomes that were subjected to analysis. The annotation of Pfam domains was carried out using Funannotate pipeline v1.8.9, with reference to the Pfam database.

**
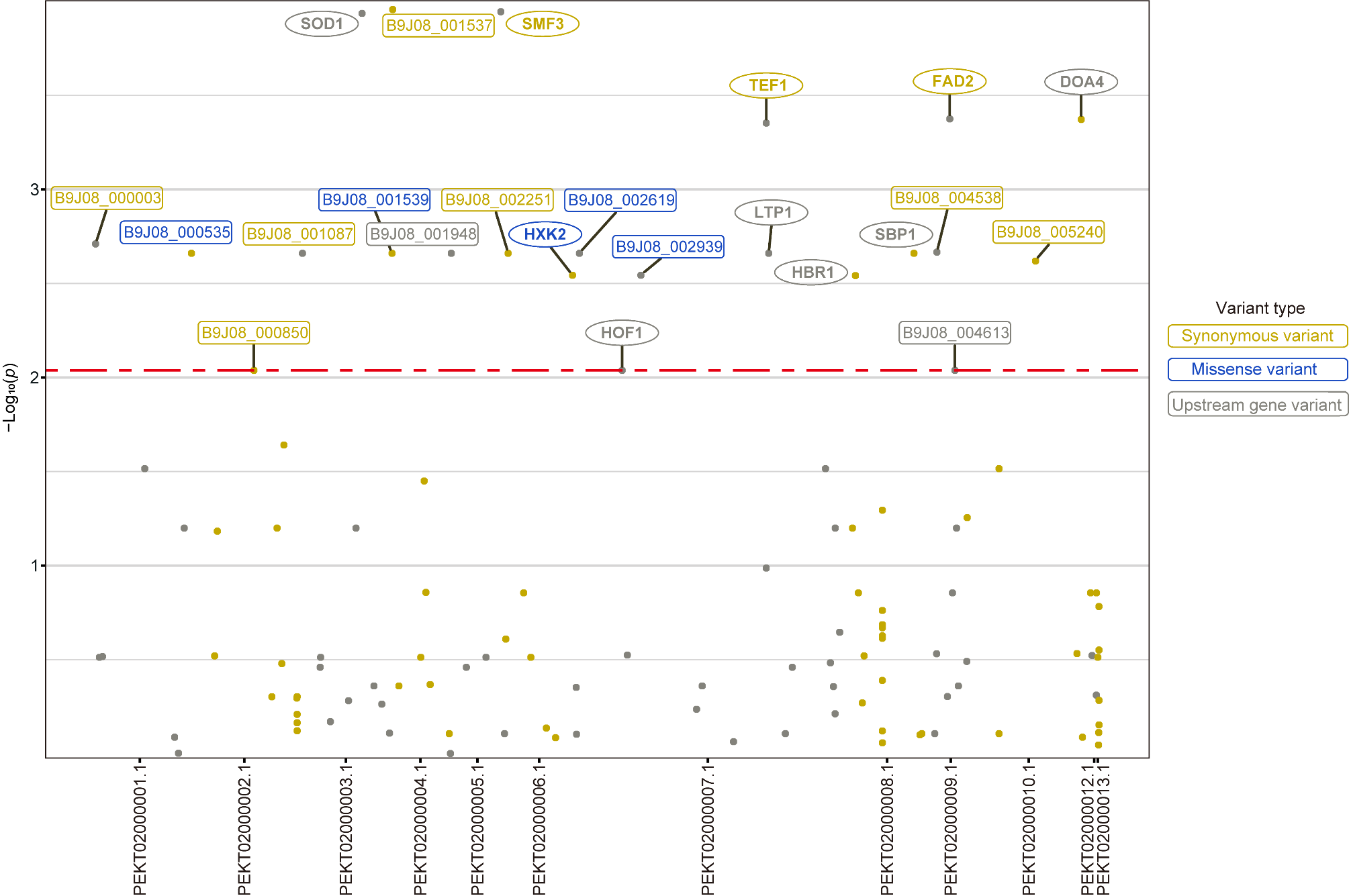
**

**Figure S2. Genome-wide association study (GWAS) of fluconazole resistance in *C. auris* Clade IV.**

The y-axis represents statistical significance as -log_10_(*p*), with the red dashed line indicating the significance threshold (*p* = 0.01). The x-axis shows genomic positions across different contigs (PEKT02000001-PEKT02000013). Significant variants are labeled with their associated genes. Variants are color-coded according to their type: blue boxes indicate missense variants, yellow boxes represent synonymous variants, and gray boxes show upstream gene variants. Key genes with potential roles in fluconazole resistance are highlighted in elliptical label boxes.

**
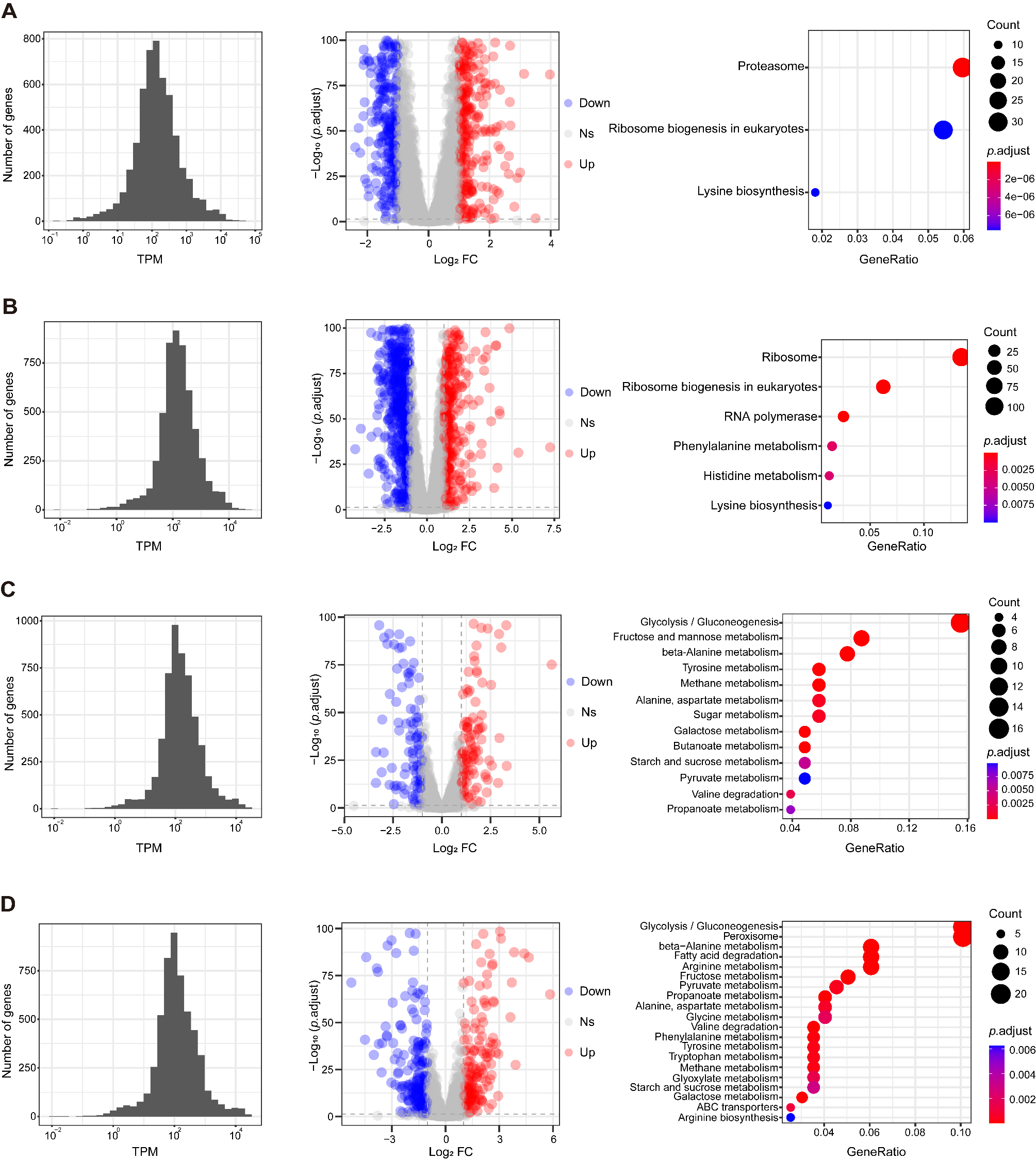
**

**Figure S3. The functional enrichment analysis of *C. auris* under indicated conditions.**

The distribution of gene counts, along with their significant regulation patterns and KEGG pathway enrichments, are presented for genes under H_2_O_2_ (A), Rapamycin (B), Glc (C), Gly (D) conditions. In volcano plots, the vertical dotted lines mark the cutoff of a two-fold change in gene expression, while the horizontal dotted line indicates the significance cutoff at *p* = 0.05. For the bubble plots, the *p* values have been adjusted using the Benjamini-Hochberg method to account for multiple hypothesis testing.

**
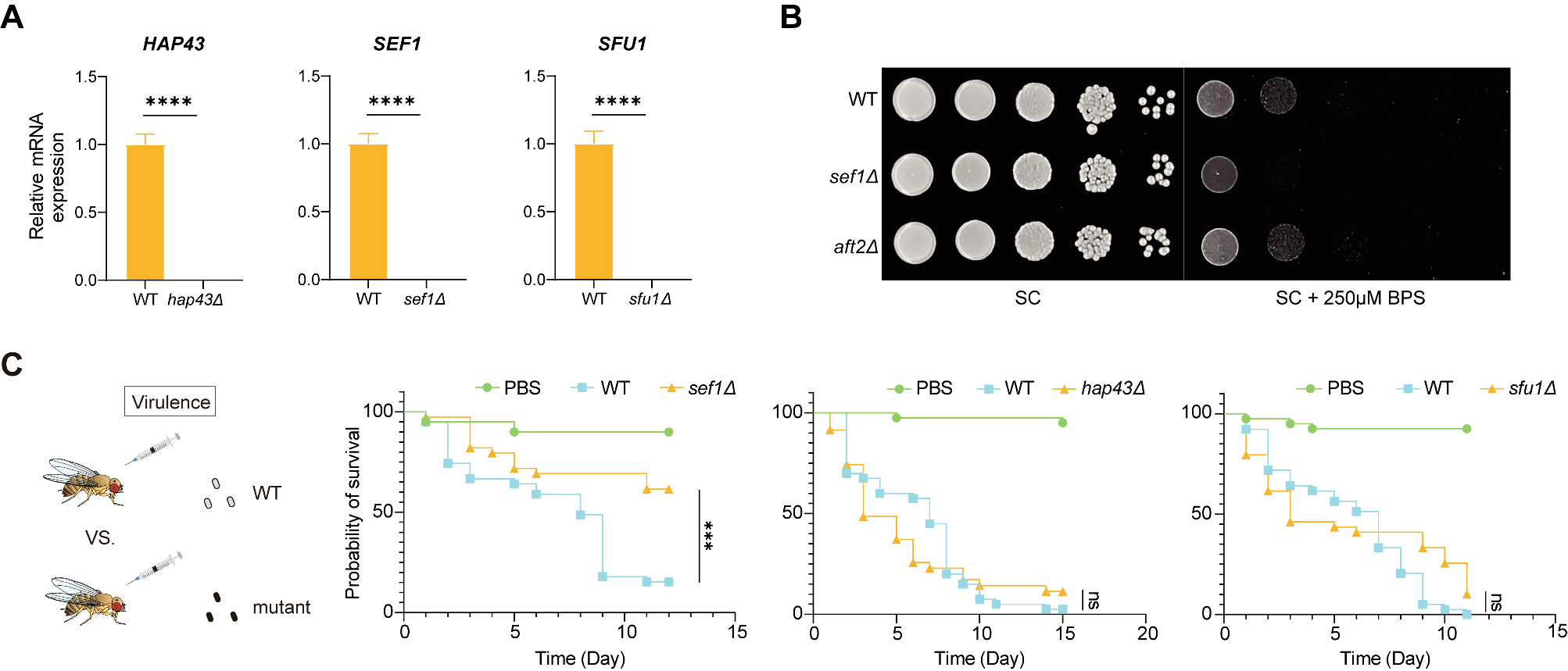
**

**Figure S4. The iron regulatory circuit differs in *C. auris* and *S. cerevisiae*.**

(A) qRT-PCR validates the gene deletion of *HAP43*, *SEF1* or *SFU1* in relevant mutant strains. **p* < 0.05, ***p* < 0.01, ****p* < 0.001, *****p* < 0.0001 by unpaired Student’s *t* test.

(B) The growth phenotype of the *C. auris* *aft2Δ* mutant cultivated on the iron-limited YPD plates was observed after an incubation time of 48 h.

(C) Virulence experiments. *Drosophila* were microinjected with individual *C. auris* strains (WT, *sef1Δ*, *hap43Δ* or *sfu1Δ*), and time to illness was monitored.

Survival data were evaluated by Kaplan-Meier analysis and statistical significance was calculated using a log rank (Mantel-Cox) test (C).

**
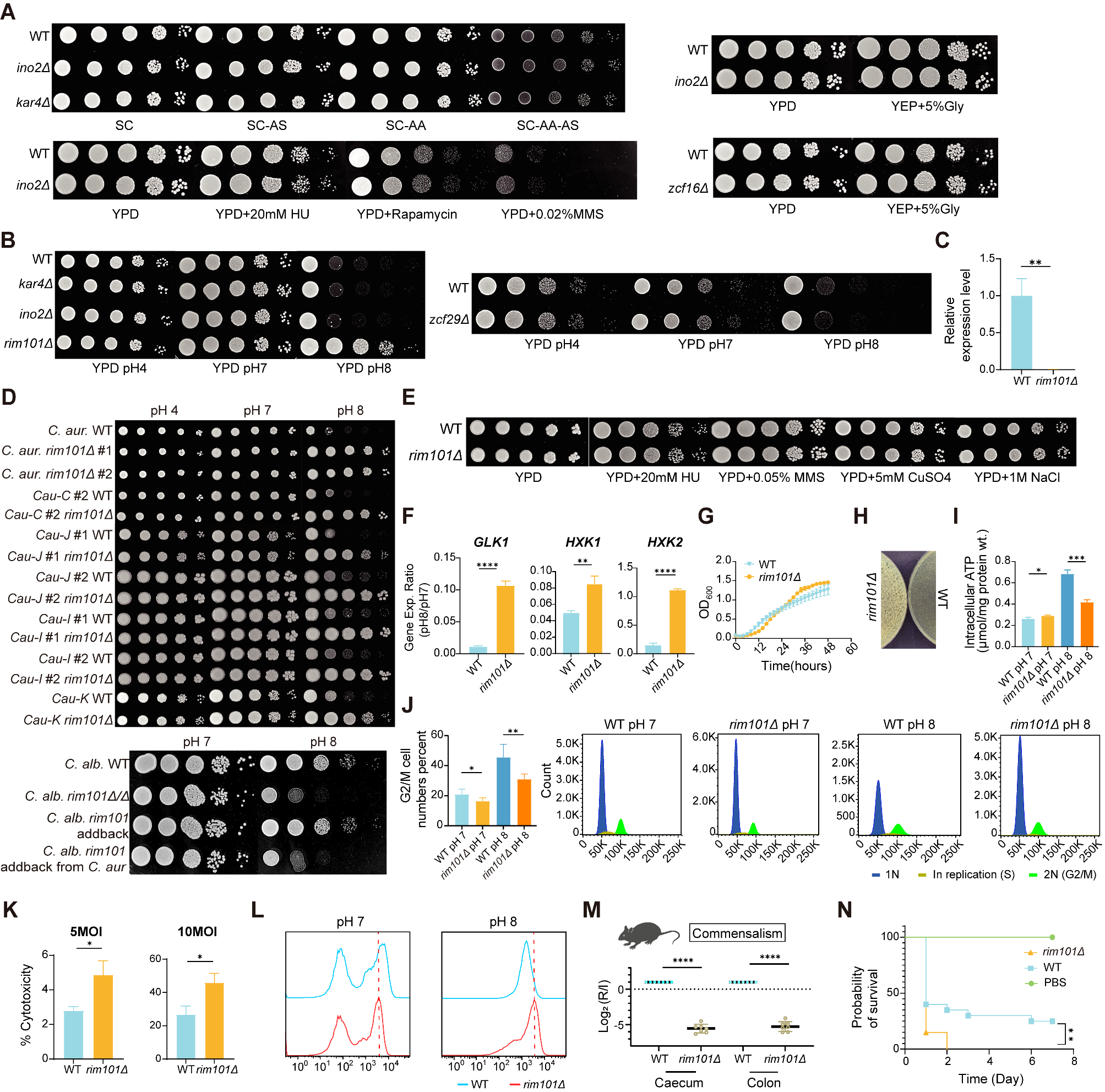
**

**Figure S5. *C. auris* gains enhanced resistance to alkaline stress through the deletion of gene *RIM101*.**

(A) The growth phenotypes of WT, *ino2Δ*, *kar4Δ* and *zcf16Δ* mutant strains grown under various stress conditions.

(B) The growth phenotypes of the deletion mutants for candidate TF genes grown on the YPD plate buffered with different pHs.

(C) qRT-PCR validates the deletion of gene *RIM101* in the putative *rim101Δ* mutant.

(D) The growth phenotype of the *rim101Δ* mutant strains, which were derived from *C. auris* isolates soured from various regions, were observed on YPD plate buffered with different pH levels after an incubation time of 48 h. Notes: one strain from China (Cau-C #2), two strains from Japan (Cau-J #1–2), two strains from India (Cau-I #1–2), and one strain from Korea (Cau-K).

(E) The growth phenotypes of the WT and *rim101Δ* mutant strains were observed under various stress conditions after an incubation time of 48 h.

(F) The expression ratio of genes *GLK1*, *HXK1* and *HXK2*, which all encode hexokinases.

(G) The growth phenotypes of the WT and *rim101Δ* mutant strains were monitored. These strains were incubated in a liquid YPD broth buffered with a pH of 8.0 at 30°C for a duration of 48 h, and OD_600_ values were detected using a BioTek Synergy H1 instrument.

(H) *C. auris* cells (2$\times$ 10^5^ CFU) that have been cultured overnight were patched onto a 10 cm solid YPD medium with a pH of 8.0. Then, the plates were incubated at 30°C for 34 h. All the fungal samples used in the animal experiments were collected under these specific incubation conditions.

(I) Measurement of intracellular ATP levels from the cells grown in (H).

(J) Cell cycle analysis assay. The fungal cells obtained from solid medium were prepared as previously described and stained with Propidium Iodide (PI), DNA content was acquired by flow cytometry, with log_2_-transformed DNA intensity values normalized to the modal value of 1N DNA. Cells were automatically sorted into 1N, S phase, and 2N populations using FlowJo software.

(K) Cytotoxicity assay. LDH concentrations released from J744 cells were detected using CytoTox 96^®^ Non-Radioactive Cytotoxicity Assay (Promega) after co-incubation with WT and *rim101Δ* strains, indicating cytotoxicity of the strains.

(L) Alterations of cell wall mannan contents in *C. auris* *rim101Δ* strain compared with WT. Fungal cells grown on neutral or alkaline solid plates were stained with ConA-FITC to visualize mannan content. The fluorescence intensity, representing the mannan levels, was quantified by flow cytometry. Data are representative of three independent and reproducible experiments.

(M) Commensalism experiment. C57 mice were infected by gavage with 1:1 mixture of the *C. auris* WT strain and *rim101Δ* strain. The abundance of each strain in the inoculum (I) and after recovery from intestinal contents (R) was determined by qPCR.

(N) Virulence experiment. *Galleria mellonella* were microinjected with 1$\times$10^6^ individual *C. auris* strains (WT or *rim101Δ*), and time to illness was monitored. Survival data were evaluated by Kaplan-Meier analysis and statistical significance was determined using the log rank (Mantel-Cox) test.

**p* < 0.05, ***p* < 0.01, ****p* < 0.001, *****p* < 0.0001 by unpaired Student’s *t* test (C, F, K, J).

**Supporting Tables**

**Table S1. Data information regarding the whole-genome sequencing of the 1,306 *C. auris* strains.**

**Table S2. Summary of the genome assembly of the 1,306 *C. auris* strains.**

**Table S3. Details of the genome assembly of the 1,306 *C. auris* strains.**

**Table S4. Gene distribution in the pan-genome of the 1,306 *C. auris* strains.**

**Table S5. Functional annotation of the pan-genome across six distinct Clades.**

**Table S6. Annotation of SNPs in the pan-genome.**

**Table S7. Description of a total of 32 experimental conditions.**

**Table S8. GSEA results for *C. auris* in comparison with other strains under six distinct stress conditions.**

**Table S9. Annotation of LncRNA.**

**Table S10. Annotation of genes within the selected cMonkey2 clusters.**

**Table S11. Strains and plasmids used in this study.**

**Table S12. Primers used in this study.**

**Table S13. Reagents used in this study.**
